# Distinct mRNA and protein interactomes highlight functional differentiation of major eIF4F-like complexes from *Trypanosoma brucei*

**DOI:** 10.3389/fmolb.2022.971811

**Published:** 2022-10-07

**Authors:** Maria J. R. Bezerra, Danielle M. N. Moura, Eden R. Freire, Fabiola B. Holetz, Christian R. S. Reis, Tallyta T. S. Monteiro, Adriana R. S. Pinto, Ning Zhang, Antonio M. Rezende, Antonio Pereira-Neves, Regina C. B. Q. Figueiredo, Christine Clayton, Mark C. Field, Mark Carrington, Osvaldo P. de Melo Neto

**Affiliations:** ^1^ Aggeu Magalhães Institute, Oswaldo Cruz Foundation, Recife, Pernambuco, Brazil; ^2^ Department of Genetics, Federal University of Pernambuco, Recife, Pernambuco, Brazil; ^3^ Carlos Chagas Institute, Oswaldo Cruz Foundation, Curitiba, Pernambuco, Brazil; ^4^ School of Life Sciences, University of Dundee, Dundee, United Kingdom; ^5^ Heidelberg University Center for Molecular Biology, Heidelberg, Germany; ^6^ Institute of Parasitology, Biology Centre, Czech Academy of Sciences, České Budějovice, Czechia; ^7^ Department of Biochemistry, University of Cambridge, Cambridge, United Kingdom

**Keywords:** protein synthesis, translation initiation, eIF4E, eIF4G, PABP, trypanosome, RNA processing

## Abstract

Gene expression in pathogenic protozoans of the family Trypanosomatidae has several novel features, including multiple eIF4F-like complexes involved in protein synthesis. The eukaryotic eIF4F complex, formed mainly by eIF4E and eIF4G subunits, is responsible for the canonical selection of mRNAs required for the initiation of mRNA translation. The best-known complexes implicated in translation in trypanosomatids are based on two related pairs of eIF4E and eIF4G subunits (EIF4E3/EIF4G4 and EIF4E4/EIF4G3), whose functional distinctions remain to be fully described. Here, to define interactomes associated with both complexes in *Trypanosoma brucei* procyclic forms, we performed parallel immunoprecipitation experiments followed by identification of proteins co-precipitated with the four tagged eIF4E and eIF4G subunits. A number of different protein partners, including RNA binding proteins and helicases, specifically co-precipitate with each complex. Highlights with the EIF4E4/EIF4G3 pair include RBP23, PABP1, EIF4AI and the CRK1 kinase. Co-precipitated partners with the EIF4E3/EIF4G4 pair are more diverse and include DRBD2, PABP2 and different zinc-finger proteins and RNA helicases. EIF4E3/EIF4G4 are essential for viability and to better define their role, we further investigated their phenotypes after knockdown. Depletion of either EIF4E3/EIF4G4 mRNAs lead to aberrant morphology with a more direct impact on events associated with cytokinesis. We also sought to identify those mRNAs differentially associated with each complex through CLIP-seq with the two eIF4E subunits. Predominant among EIF4E4-bound transcripts are those encoding ribosomal proteins, absent from those found with EIF4E3, which are generally more diverse. RNAi mediated depletion of EIF4E4, which does not affect proliferation, does not lead to changes in mRNAs or proteins associated with EIF4E3, confirming a lack of redundancy and distinct roles for the two complexes.

## Introduction

The Trypanosomatidae family of flagellated protozoa includes many different pathogenic species, some of those infecting only invertebrates and others with complex life cycles that include more than one host (Jackson et al., 2016). Trypanosomatids are members of the supergroup Excavata, which diverged from other eukaryotes some one billion years ago and have evolved or retained features not common in other eukaryotes, including polycistronic transcription of protein coding genes (reviewed in Maslov et al., 2019; Vesteg et al., 2019). Transcripts from polycistronic units are co-transcriptionally processed by *trans-*splicing into capped, monocistronic and polyadenylated mRNAs. During *trans*-splicing each mRNA receives a common 5′-end exon, the spliced-leader (SL, 39 nucleotides in length in *Leishmania* and *Trypanosoma*), which has a uniquely modified 5′ *cap*, named *cap4* (Bangs et al., 1992; Martinez-Calvillo et al., 2010; Michaeli, 2011). Co-transcription of many genes producing mRNAs and proteins with different abundances and expression patterns means that most regulation of individual gene expression occurs through post-transcriptional mechanisms: mRNA maturation, mRNA half-life, translation efficiency and protein stability (Clayton, 2019; Karamysheva et al., 2020).

Translation initiation is an important site for regulation of protein synthesis in eukaryotes (Ali et al., 2017; Genuth and Barna, 2018). It usually starts with the eIF4F complex binding the 5′ cap of the mRNA. In mammals, eIF4F contains three subunits: eIF4E, which binds the m^7^GTP cap; eIF4G, a large polypeptide that acts as a scaffold, mediates interaction with a number of protein partners; and eIF4A, an RNA helicase that remodels mRNA secondary structures in the presence of ATP (Merrick, 2015; Hernández et al., 2020; Romagnoli et al., 2021). Similar eIF4F complexes are present in plants (Browning and Bailey-Serres, 2015; Castellano and Merchante, 2021), while in yeast eIF4F has been described as being constituted mainly of the eIF4E and eIF4G subunits alone (Lanker et al., 1992; Merrick, 2015), likely reflecting a looser association of eIF4A. Binding of eIF4F to the 5′ cap facilitates the recruitment of the small ribosomal subunit and other translation initiation factors, including the large eIF3 complex, to the mRNA. The small subunit then scans the mRNA 5′ untranslated region (UTR) until encountering the AUG initiation codon, a step that precedes the proper start of translation. eIF4F also binds to the poly-A binding protein (PABP), which itself binds the 3’ poly-A tail of the mRNA and can effectively circularize the mRNA and enhance subsequent rounds of translation (Hinnebusch, 2014; Shirokikh and Preiss, 2018).

While the ubiquitous presence of the eIF4F complex suggests that it arose before the last eukaryotic common ancestor, subsequently components have undergone expansions and/or divergence. For example, in the trypanosomatids there are six homologues of eIF4E (EIF4E1 to EIF4E6) and five of eIF4G (EIF4G1 to EIF4G5), all simultaneously expressed and none closely related to those found in better known eukaryotes (Dhalia et al., 2005; Freire et al., 2014a, 2014b; Tinti and Ferguson, 2022). In contrast, there is only a single eIF4A involved in translation (Dhalia et al., 2006). Until recently, the only two eIF4F complexes clearly implicated in translation initiation were those containing the pairings of EIF4E4 to EIF4G3 in one and EIF4E3 to EIF4G4 in the other. In *Trypanosoma brucei,* both sets of subunits were associated with polysomes, enhanced expression of a reporter mRNA in a tethering assay and are essential for proliferation in the developmental form present in mammals (bloodstream form: BSF). In contrast, there is evidence that EIF4E4 is not required for viability in the cultured developmental form from the tsetse fly midgut (procyclic culture form: PCF). However, these observations were made following RNAi mediated silencing as opposed to gene deletion (reviewed in Freire et al., 2017). RNAi followed by metabolic labelling has also implied a role for all four proteins during translation (Freire et al., 2011; Moura et al., 2015). In a second trypanosomatid lineage, *Leishmania* sp., both EIF4E4/EIF4G3 and EIF4E3/EIF4G4 complexes are associated with the eIF3 initiation complex (Zinoviev et al., 2012b; Meleppattu et al., 2015), further evidence for both being involved in translation initiation. Recently, indirect evidence implicated the EIF4E6/EIF4G5 complex in translation (Melo do Nascimento et al., 2020, 2021).

There has also been expansion of PABP paralogues in trypanosomatids, with three in *Leishmania* (PABP1 to PABP3) and two in *T. brucei* (PABP1 and PABP2) (Kramer et al., 2013). PABP1 binds mRNA populations distinct from those associated with either PABP2/PABP3 or PABP2 (da Costa Lima et al., 2010; Assis et al., 2021). In both *Leishmania* and *T. brucei*, PABP1 specifically co-precipitate EIF4E4 and EIF4G3, whereas *T. brucei* PABP2 co-precipitates a wider range of different eIF4E/eIF4G subunits (de Melo Neto et al., 2018; Zoltner et al., 2018). Importantly, *Leishmania* PABP1 interacts directly with EIF4E4: PAM2 motifs (12 amino acids in length and present in several PABP interacting proteins), within the unique N-terminus of EIF4E4, interact with the C-terminal, α-helical, MLLE domain of PABP1 (de Melo Neto et al., 2015; 2018; dos Santos Rodrigues et al., 2018).

Trypanosomatid species have undergone more than one hundred million years of separate evolution, and conservation of multiple eIF4E and eIF4G homologues (Bannerman et al., 2018) implies discrete functions in translation, and potentially novel mechanisms. However, no clearly defined functional distinctions have been found between the two most closely related complexes, based on EIF4E4/EIF4G3 and EIF4E3/EIF4G4. Studies in *Leishmania* identified the eIF4E and eIF4G pairings and associated PABP1 but no clearly defined specific protein partners could be assigned to either complex (Zinoviev et al., 2011, 2012b; Shrivastava et al., 2019a). Here we advance understanding of both EIF4E3/EIF4G4 and EIF4E4/EIF4G3 complexes through the identification of known and novel protein partners associated with both sets of subunits, as well as mRNA subpopulations specifically co-precipitated with EIF4E3 or EIF4E4. Possible functional redundancies were investigated after RNAi mediated depletion. Our study defines specific partners associated with each complex as well as different mRNA targets which may be associated with distinct modes of translational regulation.

## Materials and methods

### Cell culture and transfection

Procyclic *T. brucei* Lister 427 cells were cultured at 27°C in SDM-79 medium supplemented with fetal calf serum, haemin and antibiotics. Cultures were grown to mid-log phase for all procedures. For tagging the N-terminus of proteins by modifying the endogenous loci, transfections were performed as described previously (Hill et al., 1999) using PCR products consisting of a blasticidin resistance cassette followed by an enhanced Yellow Fluorescent Protein (eYFP) tag plus 80 bp targeting sequences at each end of the PCR product (list of primers in Supplementary Table S1). Trypanosomes are diploid and the remaining wild type allele was deleted by homologous replacement with an antibiotic resistance open reading frame. For the RNAi experiments, the *T. brucei* Lister 427 cell line expressing pSMOx (Poon et al., 2012) was used with p2T7-177-derived plasmids for depletion of EIF4E3, EIF4E4 or EIF4G4, as previously described (Freire et al., 2011; Moura et al., 2015). RNAi induction was performed by addition of 1 μg/ml tetracycline or doxycycline and all results shown are representative of at least three independent experiments.

### Western-blot methods and antibodies

Transfected cell lines were tested for eYFP-tagged protein expression and depletion after RNAi induction by western blotting. Cells were harvested and washed with SDM-79 medium without serum followed by resuspension of the cell pellet directly in SDS-PAGE sample buffer and heating for 5 min at 100°C. The cell extracts were resolved on 15% SDS-PAGE gels, transferred to a PVDF membrane and probed using affinity purified rabbit antisera for EIF4E3 or EIF4E4 or EIF4G3 or EIF4G4, all at 1:2000 dilutions (Freire et al., 2011; Moura et al., 2015) and HRP-conjugated goat anti-rabbit IgG (Jackson Immunoresearch) was used as the secondary antibody.

### Immunoprecipitation and mass spectrometry analysis

For initial identification of proteins that co-precipitated with the target complexes we used a set of procyclic cell lines expressing N-terminal tagged EIF4E3^eYFP/-^, EIF4E4^eYFP/-^, EIF4G3^eYFP/+^ and EIF4G4^eYFP/+^. The cells were grown to mid log phase, harvested in 100 mM KCl, 1 mM MgCl_2_, 50 mM Hepes pH 7.2, 5% glycerol, then disrupted using cryogenic grinding and purified as previously described (Field et al., 2012), using nanobodies from llama (clone LaG-16–G4S–LaG-2) coupled to Dynabeads M-270 epoxy (Invitrogen). Lysates from a cell line expressing the nuclear protein eYFP-DRBD4 (Tb927.11.14100) was used as a negative control. In a second approach, in order to evaluate whether EIF4E3 bound additional or alternative proteins in the absence of EIF4E4 and vice-versa, RNAi was induced for 48 h and cells were lysed by cavitation (Mureev et al., 2009), with IPs then performed using specific polyclonal antibodies (anti-EIF4E3 and anti-EIF4E4) coupled to Protein A Dynabeads (Invitrogen), following the manufacturer’s instructions. This generated the samples EIF4E3/RNAi EIF4E4 and EIF4E4/RNAi EIF4E3.

For all IPs, immunoprecipitated proteins were eluted directly into SDS-PAGE sample buffer and loaded unto denaturing SDS-PAGE gels, with the electrophoresis interrupted immediately after the proteins entered the resolving gel. After brief Coomassie Blue staining, the whole set of proteins was excised and submitted to mass spectrometry analyses in a LTQ Orbitrap XL EDT Thermo Scientific. All reported IPs were performed in biological triplicates, with the mass spectrometry data essentially normalized as previously described (Assis et al., 2021). For the IPs with the eYFP-tagged baits, for instance, a first normalization was performed considering the sum of all intensities for each of the samples analyzed together (15 samples considering the four proteins investigated plus the control) and using the highest sum to normalize the remaining samples. For each co-precipitated polypeptide, the averages from the normalized intensities were then calculated from the three replicates of each tagged protein. These averages were then used to calculate the enrichment ratio between each tagged eIF4E or eIF4G homologue and the negative control. A similar approach was used to process the samples from the RNAi experiment.

The mass spectrometry proteomics data have been deposited to the ProteomeXchange Consortium *via* the PRIDE (Perez-Riverol et al., 2019) partner repository with the following dataset identifier: PXD033857. Reviewer account details are: username—reviewer_pxd022480@ebi.ac.uk; password -Lmg0kVPT.

### Microscopy analysis

Procyclic-form RNAi-induced cells, and non-induced controls, were harvested, washed with PBS, stained with DAPI and then directly observed using Zeiss LZSM510 META confocal microscope. Parasites submitted to these same experimental conditions were further analysed using transmission electron microscopy. The cells were fixed in 2.5% glutaraldehyde in 0.1 M cacodylate buffer, pH 7.2, followed by post fixation for 30 min in 1% OsO_4_ in 0.1 M cacodylate buffer, dehydration in increasing concentrations of acetone and embedding in Epon (Polybed 812) resin. Ultra-thin sections were harvested on 300 mesh copper grids, stained with 5% uranyl acetate and 1% lead citrate, and observed with a FEI Tecnai Spirit transmission electron microscope. The images were randomly acquired with a CCD camera system (MegaView G2, Olympus, Germany).

### RNA co-precipitation and sequencing methods

For the cross-linking, immunoprecipitation and RNA sequence (CLIP-seq), 2.5 × 10^9^ EIF4E3 or EIF4E4 RNAi cells were induced for 48 h and exposed to UV crosslinking with three pulses of 300 mJ/cm^2^ UV light for 1 minute each. Non-induced cells were used as a control. Cells were then harvested and lysed using cavitation (Mureev et al., 2009). Targeted proteins were immunoprecipitated using Dynabeads (Invitrogen) coupled with rabbit immunopurified polyclonal antibodies produced against EIF4E3 or EIF4E4. Co-precipitated RNAs were extracted with the RNAeasy extraction Kit (Qiagen) and quantified on a Qubit 2.0 fluorochemical apparatus (Thermo Fisher) with the HS Assay Kit RNA reagent (Thermo Fisher). cDNA libraries were made with the TruSeq Stranded mRNA Library Prep Kit system (Illumina Inc.) and validated using the KAPA Library Quantification Kit and by agarose gel. Sequencing was carried out in the MiSeq platform with the MiSeq Reagent Kit v3 cartridge 150 cycles (Illumina), with the data analyzed by FastQC to check for quality, followed by removal of low-quality sequences (Phred <20 and size smaller than 40 pb) using Trimmomatic 0.36 (http://www.usadellab.org/cms/?page=trimmomatic). The STAR program (Dobin et al., 2013) was used to map the sequences to the *T. brucei* genome (TriTrypDB-40_TbruceiTREU927_Genome.fasta) and to count the number of reads. The identification of differentially expressed genes was performed using number of transcripts per million as the measure of transcript abundance (Wagner et al., 2012). A minimum of 50 reads per transcript value (average of three biological replicates) were used and a minimum of 2-fold enrichment over the negative control (Log_2_ (I Ratio) >1) was considered. The sequenced data has been deposited at https://www.ncbi.nlm.nih.gov/geo/ with the following accession number: GSE206064.

For the SOLiD RNA-seq analysis, immunoprecipitations and RNA extractions were performed as previously described (Holetz et al., 2010). In brief, *T. brucei* cytoplasmic extracts (equivalent to 1 × 10^9^ cells) were incubated with approximately 0.1 mg of protein A sepharose beads previously linked to affinity purified polyclonal antibodies raised against EIF4E3 or EIF4E4, for 1 h, at 4°C, in the presence of 200 U/ml of RNAseOUT. As a control, cytoplasmic extracts were incubated with the beads in the absence of antibodies. Beads were washed three times with IPM2 buffer (KCl, 100 mm; MgCl_2_, 5 mm; Hepes, 10 mm, pH 7.0; protease inhibitors, 1:100; RNase OUT, 200 U/ml); Nonidet P40, 1%). Co-precipitated RNAs were extracted with the RNAeasy extraction Kit (Qiagen) as described above. The experiments were performed in triplicate and the immunoprecipitated RNA fraction was analyzed by deep sequencing on a Life Technologies SOLiD4 equipment. The data were analyzed using CLC Genomics Workbench 5, and the reads trimmed on the basis of quality, using a threshold phred score of 15. The genome used for mapping was the TriTrypDB v. 4.0 (Tbrucei427Genomic_TriTrypDB-4.0. fasta) and the alignment performed with the following parameters: additional upstream and downstream sequences of 100 bases; minimum number of reads, 10; maximum number of mismatches, 2; nonspecific match limit, -2. We selected possible targets of EIF4E3 and EIF4E4 with the β binomial statistical test (Baggerly’s test) with a *p-value* corrected FDR of ≤1%, a minimum RPKM value of 50 and considering a minimum of 2-fold enrichment over the negative control (Log_2_ (I Ratio) >1).

## Results

### Single alleles of EIF4E3 and EIF4E4 support cell growth but both copies of EIF4G3 and EIF4G4 are required

**FIGURE 1:**
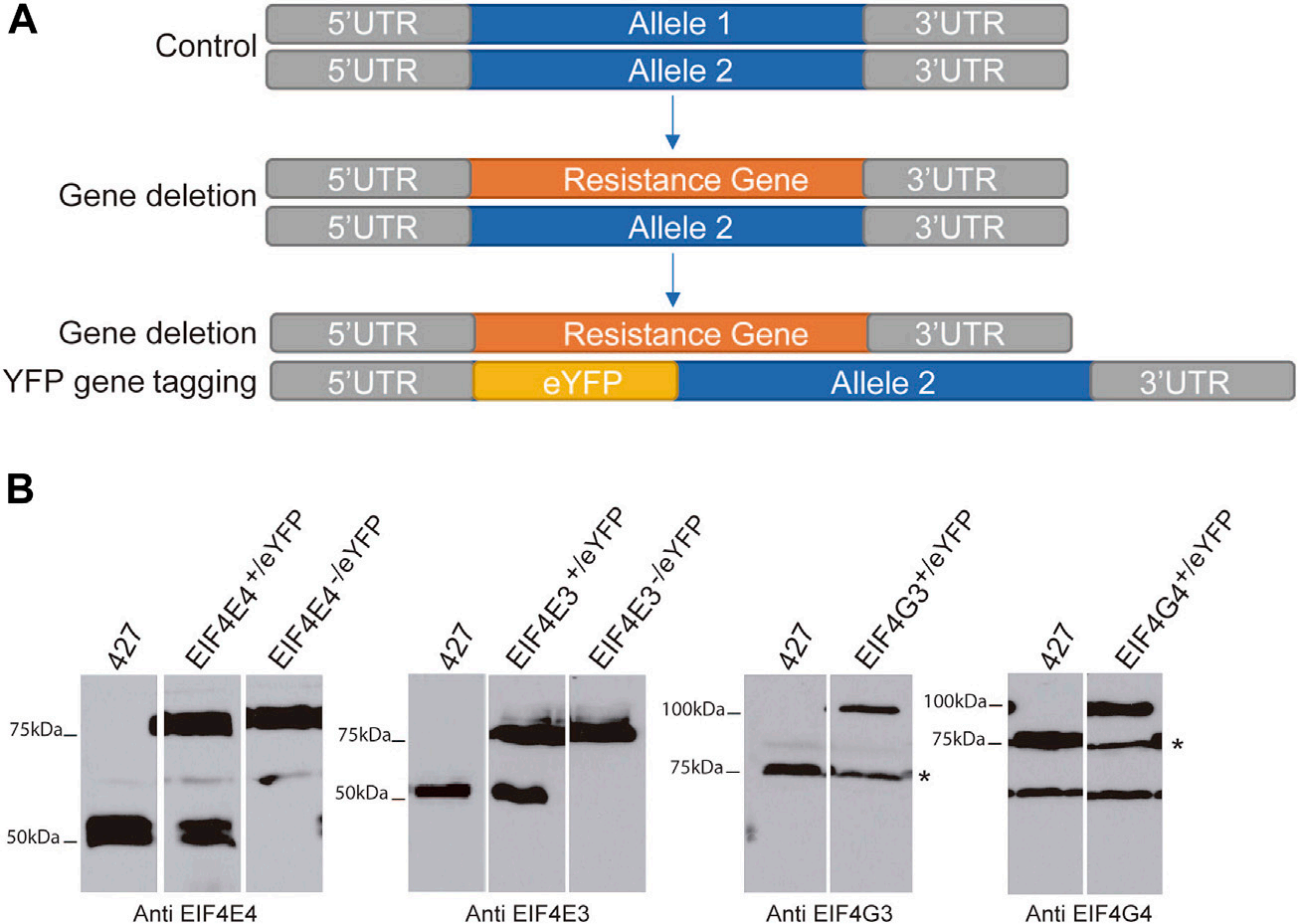
Expression of eYFP-tagged EIF4E3, EIF4E4, EIF4G3 and EIF4G4 proteins. **(A)** Representative scheme of eYFP-tag addition in target genes. **(B)** Immunodetection of the four eYFP-tagged proteins using cytoplasmic extracts from the various *T. brucei* cell lines generated as well as from cells lacking eYFP-tagged proteins (427—Lister 427 procyclic cells). The asterisk for the eIF4G blots indicates the endogenous EIF4G3 and EIF4G4 bands. For EIF4G4, a non-specific band of lower molecular weight, a product of cross-reaction with the antibodies used, is also seen. All the separated lanes shown in the different panels are from the same blot and with the same exposure. Numbers on the left indicate the sizes of molecular weight markers (in kiloDaltons).

To systematically investigate the composition of the *T. brucei* EIF4E3/EIF4G4 and EIF4E4/EIF4G3 complexes and identify differences which may reflect distinct functional properties, we first sought to identify further proteins associated with the two complexes. To achieve this, we performed parallel experiments with both eIF4E and both eIF4G subunits from each complex. Each protein was tagged at the N-terminus with enhanced yellow fluorescent protein (eYFP) by modification on an endogenous allele. Trypanosomes are diploid and for the two eIF4Es, the second allele was deleted leaving cells with only a tagged eIF4E (Figure 1A). The absence of the native EIF4E3/EIF4E4 proteins was confirmed by western blot analysis using antibodies against the corresponding native proteins (Figure 1B). The resulting single allele EIF4E3^-/eYFP^ and EIF4E4^-/eYFP^ cell lines proliferated at the same rate as wild type cells. For the eIF4Gs, one allele was successfully modified to express an eYFP tagged protein. However, after several gene deletion attempts using two different selectable markers, deletion of the second allele could not be achieved. The same occurred when deletion of a single allele of either eIF4G was attempted in wild type cells; no cell lines with a single deletion were recovered. These observations are compatible with the eYFP-tagging of both eIF4Es not affecting function, but they also suggest haplo-insufficiency for both eIF4G homologues, with the tagging also not significantly interfering with their functions. Western blotting confirmed the expression of the tagged eIF4E proteins in the transfected cell lines and the absence of the native proteins (Figure 1B). However, in the case of eIF4Gs, the presence of the tagged protein led to a reduction in levels of the corresponding native protein. A reduction in native EIF4G3 and EIF4G4 levels has also been seen previously after ectopic expression of these TY-tagged proteins (Moura et al., 2015), reinforcing a strict requirement regarding the levels of the two eIF4G homologues. For EIF4G3, at least, this is also compatible with the RNAi depletion experiments where minor reductions in levels were associated with extensive growth inhibition, indicating a lack of tolerance to changes in abundance. Quantitation of the levels of EIF4E4 and EIF4G3 found them to be roughly equivalent, with EIF4E3 estimated to be 10-fold more abundant than EIF4G4 (Moura et al., 2015). These differences in levels between EIF4E3 and EIF4G4 are reminiscent of those seen between the yeast eIF4E/eIF4G1 pair (von der Haar and McCarthy, 2002), although recent quantitative mass spectrometry results suggest that the two *T. brucei* proteins may be also present at similar levels (Tinti and Ferguson, 2022).

### Defining the interactomes for the EIF4E4/EIF4G3 and EIF4E3/EIF4G4 complexes

To identify proteins associated with the EIF4E4/EIF4G3 and EIF4E3/EIF4G4 complexes, we prepared lysates by cryo-milling cells from the cell lines expressing the eYFP tagged proteins and used anti-GFP-antibody-coupled beads to immunoprecipitate the tagged baits and associated proteins. We deliberately did not add RNAse in order to maintain complexes or associations which required the presence of RNA. As a negative control, a cell line expressing an eYFP-tagged nuclear protein, DRBD4 (Tb927.11.14100), was used. After immunoprecipitation and elution (Supplementary Figure S1), all samples were submitted for mass spectrometry analysis. Normalized intensities derived from the various co-precipitated proteins from sets of three replicates, and with a minimum of two peptides found in at least two replicates, were then used to generate lists of proteins enriched 2-fold or more with each tagged protein in comparison to the negative control (Supplementary Table S2). Overall, a much larger set of co-precipitated proteins were enriched with the two eIF4G homologues (196 with EIF4G3; 52 with EIF4G4) when compared to their corresponding eIF4E partners (20 with EIF4E3; 51 with EIF4E4). This was expected due to the known roles of eIF4Gs in mediating protein-protein interactions. Nevertheless, the EIF4E4/EIF4G3 subunits were also generally enriched with a larger number of proteins than their counterparts from the EIF4E3/EIF4G4 complex.

**FIGURE 2:**
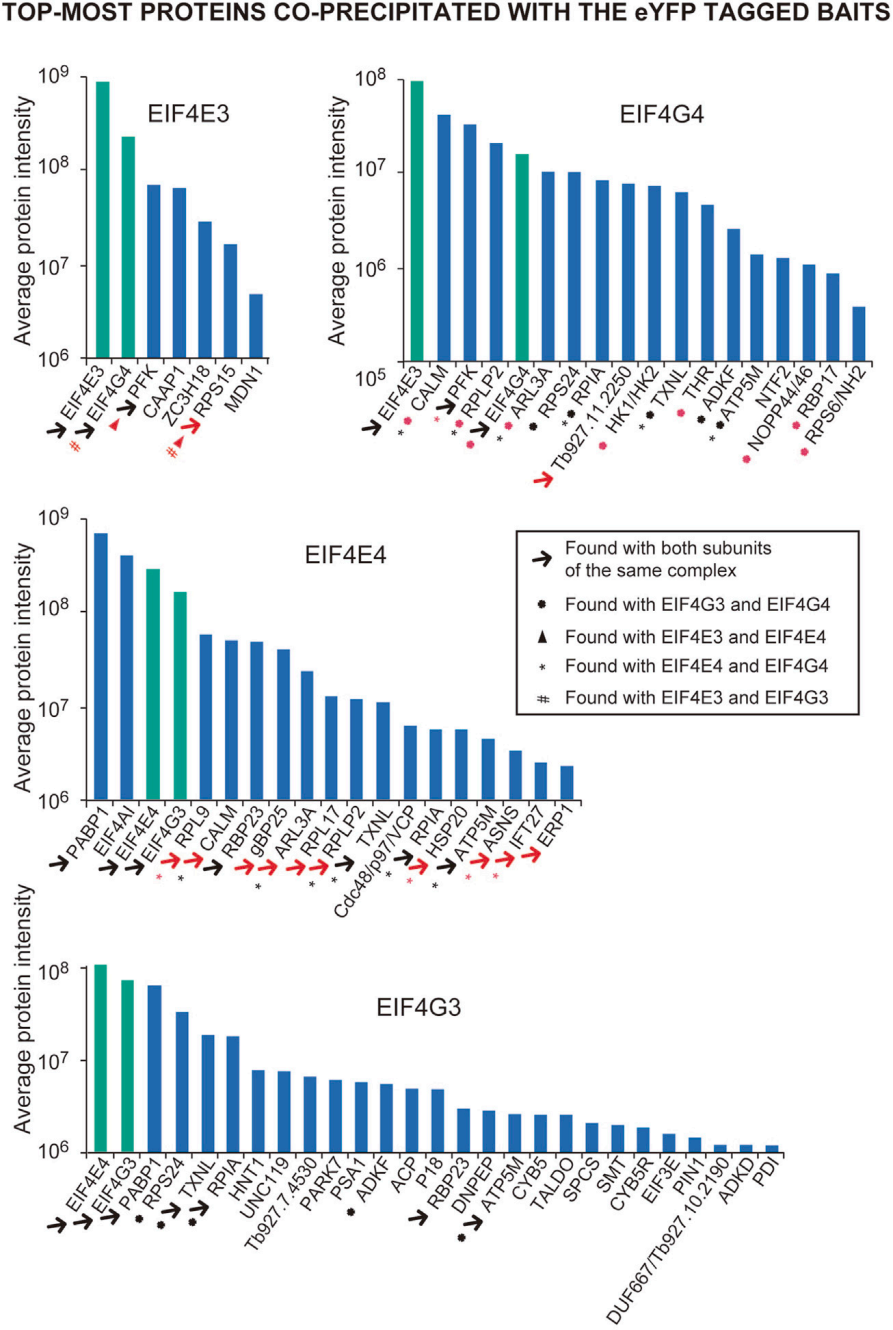
Overview of the top-most proteins co-precipitated with the eYFP tagged EIF4E3, EIF4E4, EIF4G3 and EIF4G4 in Trypanosoma brucei procyclic cells. Bar charts highlighting the proteins listed in Tables 1–4 and with enrichments relative to the negative control > 4-fold (for EIF4E3, EIF4E4 and EIF4G4) or >8-fold and with intensities greater than 10^6^ (for EIF4G3). The four proteins directly investigated here are highlighted by the green bars. The different symbols are indicated within the box, with symbols colored in black generally indicating that the proteins co-precipitating with the corresponding pair are among those shown in Tables 1–4, while the red color indicates those that are also present with the protein pair with a lower enrichment (<4 and >1.5; shown in the Supplementary Tables S2–S5). The differences in intensities seen for the four baits may, to some extent, reflect differences in abundance between them.

To facilitate the identification of co-precipitated proteins functionally relevant for the EIF4E3/EIF4G4 pair, as well as for EIF4E4, those with enrichment ratios greater than 4-fold relative to the negative control were grouped according to putative functions, as shown in Tables 1–3. For EIF4G3, since the number of proteins co-precipitating was higher, only the top-most enriched proteins with an enrichment ratio greater than 8-fold are shown in Table 4. A direct comparison of the same sets of proteins is shown in Figure 2. The mass spectrometry results confirm the co-precipitation of the two pairs of eIF4E and eIF4G subunits, consistent with the two distinct eIF4F complexes previously reported (see Introduction). For the EIF4E3/EIF4G4 complex, both subunits were among those most enriched for the corresponding partner (Figure 2, Tables 1, 2). However, none of the six co-precipitated proteins substantially enriched with EIF4E3 (>4-fold) were also found enriching with EIF4G4 only. Three of those were found only with EIF4E3: the cytoskeleton assembly protein (CAAP1), a Zinc-Finger RNA-binding protein (ZC3H18) and a nuclear protein involved in the biosynthesis of the 60S large ribosomal subunit (Midasin or MDN1). The three others, EIF4G4 as well as the enzyme phosphofructokinase and the ribosomal protein RPS15, were also found, albeit with lower enrichments, with EIF4E4 and/or EIF4G3. Indeed, even when the whole set of proteins with lower enrichments are considered (based on the data from the Supplementary Table S2), only two proteins co-precipitated with EIF4E3/EIF4G4 only: the uncharacterized Tb927.11.2250 and the zinc-finger ZC3H21. Seventy-five percent (15/20) of the proteins enriched with EIF4E3 with >2-fold were not found enriched with any of the other tagged proteins. In contrast, less than 20% (10/52) of the proteins enriched for EIF4G4 were exclusively enriched with this subunit, with the most enriched protein being an uncharacterized protein having a Nuclear Factor 2 domain (NTF2; Tb927.10.2240). Remarkably, more than 70% (38/52) of the proteins enriched with EIF4G4 are also enriched with EIF4G3 and ∼31% (16/52) also with EIF4E4.

**TABLE 1:** Most enriched proteins co-precipitated with the eYFP-tagged EIF4E3 in *Trypanosoma brucei* procyclic cells.

TriTrypDB Accession	Protein ID	Name	Description	Protein Intensity	Ratio
**Bait**					
Tb927.11.11770	Q383A4	EIF4E3	Eukaryotic translation initiation factor 4E-3	8 × 10^8^	653.25
**Translation initiation factors**					
Tb927.11.10560	Q383M3	EIF4G4	Eukaryotic translation initiation factor 4G type 4	2.1 × 10^8^	172.19
**Ribosomal Proteins**					
Tb927.7.2340	Q57XN7	RPS15	40S ribosomal protein S15	1.6 × 10^7^	4.14
**RNA binding proteins**					
Tb927.7.2140	D6XKD1	ZC3H18	Zinc finger protein family member	2.7 × 10^7^	6.65
**Intracellular transport and cell motility**					
Tb927.10.1450	Q38C95	CAAP1	Centrin arm-associated protein 1	6.2 × 10^7^	7.2
**Enzyme**					
Tb927.3.3270	D6XDN4	PFK	ATP-dependent 6-phosphofructokinase	6.6 × 10^7^	6.37
Tb927.1.880	Q4GZD0	MDN1	Midasin	4.8 × 10^6^	15.84

The table lists proteins co-immunoprecipitated with eYFP-EIF4E3 with an enrichment ratio >4 in comparison to the negative control, eYFP-DRBD4, and classified according to selected functional categories (defined in bold). The ratio column represents the average enrichment ratios.

**TABLE 2:** Most enriched proteins co-precipitated with the eYFP-tagged EIF4G4 in *Trypanosoma brucei* procyclic cells.

TriTrypDB Accession	Protein ID	Name	Description	Protein Intensity	Ratio
**Bait**					
Tb927.11.10560	Q383M3	EIF4G4	Eukaryotic translation initiation factor 4 gamma 4	2.2 × 10^7^	17.36
**Translation initiation factors**					
Tb927.11.11770	Q383A4	EIF4E3	Eukaryotic translation initiation factor 4E-3	1.4 × 10^8^	117.66
**Ribosomal Proteins**					
Tb927.9.5690	Q38EY6	RPLP2	60S acidic ribosomal protein P2	2.9 × 10^7^	4.51
Tb927.10.7340	Q38AM9	RPS24	40S ribosomal protein S24	1.4 × 10^7^	7.42
Tb927.9.5150	Q38F38	RPS6/NH2	40S ribosomal protein S6	4.1 × 10^5^	∞
**RNA binding proteins**					
Tb927.8.750	Q57YF6	NOPP44/46-2	Nucleolar RNA-binding protein	1.2 × 10^6^	5.25
Tb927.6.4530	Q587B9	RBP17	RNA-binding protein 17	9.8 × 10^5^	∞
**Intracellular transport and cell motility**					
Tb927.11.13020	Q382N3	CALM	Calmodulin	6 × 10^7^	7.04
**Enzymes**					
Tb927.3.3270	D6XDN4	PFK	ATP-dependent 6-phosphofructokinase	4.7 × 10^7^	4.49
Tb927.3.3450	Q57XJ5	ARL3A	ADP-ribosylation factor-like protein 3A	1.4 × 10^7^	4
Tb927.11.8970	Q384D1	RPIA	Ribose 5-phosphate isomerase	1.1 × 10^7^	14.08
Tb927.10.2010/Tb927.10.2020	Q38C41/Q38C42	HK1/HK2	Hexokinase	9.5 × 10^6^	4.3
Tb927.7.4520	Q57X68	TXNL	Thioredoxin-like	8.1 × 10^6^	7.45
Tb927.7.4390	Q57X55	THR	Threonine synthase	5.8 × 10^6^	5.63
Tb927.10.2530	Q38BZ2	ADKF	Adenylate kinase	3.1 × 10^6^	5.6
Tb927.7.840	Q57VT0	ATP5M	Mitochondrial ATP synthase subunit	1.6 × 10^6^	6.9
**Uncharacterized Proteins**					
Tb927.11.2250	Q386T4	CP2250	Conserved protein, unknown function *	10^7^	4.78
Tb927.10.2240	Q38C20	NTF2	Nuclear transport factor 2 (NTF2) domain protein *	1.5 × 10^6^	9.86

The proteins are listed as described for Table 1. The symbol ∞ is used for those proteins where the enrichment ratios could not be defined due to their intensities in the control samples (DRBD4) being zero. The symbol * indicates uncharacterized proteins found to stimulate expression in a tethering assay (Erben et al., 2014).

**TABLE 3:** Most enriched proteins co-precipitated with the eYFP-tagged EIF4E4 in *Trypanosoma brucei* procyclic cells.

TriTrypDBAccession	Protein ID	Name	Description	Protein Intensity	Ratio
**Bait**					
Tb927.6.1870	Q585M4	EIF4E4	Eukaryotic translation initiation factor 4E-4	2.4 × 10^8^	42.94
**Translation initiation factors**					
Tb927.9.9290	Q38E63	PABP1	Polyadenylate-binding protein	5.6 × 10^8^	78.57
Tb927.9.4680	Q38F76	EIF4AI	Eukaryotic initiation factor 4A-1	3.3 × 10^8^	9.17
Tb927.8.4820	Q57UX0	EIF4G3	Eukaryotic translation initiation factor 4 gamma 3	1.4 × 10^8^	120.96
**Ribosomal Proteins**					
Tb927.10.14580	Q385T6	RBL17	60S ribosomal protein L17	1.2 × 10^7^	11.53
Tb927.6.5120	Q586I4	RPLP2	60S acidic ribosomal protein P2	1.1 × 10^7^	5.32
Tb927.10.1100	Q38CD0	RPL9	60S ribosomal protein L9	5.1 × 10^7^	4.03
**RNA binding proteins**					
Tb927.10.11270	Q389L2	RBP23	RNA-binding protein 23	4.2 × 10^7^	124.04
Tb927.11.13280	Q382K8	gBP25	Guide RNA binding protein 25	3.6 × 10^7^	7.92
**Intracellular transport and cell motility**					
Tb927.3.3450	Q57XJ5	ARL3A	ADP-ribosylation factor-like protein 3A	2.1 × 10^7^	6.14
Tb927.3.5550	Q580S0	IFT27	Intraflagellar transport protein 27	2.4 × 10^6^	6.57
Tb927.11.15230	Q381R2	ERP1	Emp24-related protein 1	2.2 × 10^6^	16.06
**Enzymes**					
Tb927.11.13020	Q382N3	CALM	Calmodulin	4.4 × 10^7^	5.17
Tb927.11.8970	Q384D1	RPIA	Ribose 5-phosphate isomerase	5.3 × 10^6^	6.74
Tb927.7.4520	Q57X68	TXNL	Thioredoxin-like	10^7^	9.27
Tb927.7.840	Q57VT0	ATP5M	Mitochondrial ATP synthase subunit	4.2 × 10^6^	18.22
Tb927.7.1110	Q57WT9	ASNS	Asparagine synthetase a	3.2 × 10^6^	4.49
**Chaperones**					
Tb927.10.5770	Q38B27	VCP/CDC48	Valosin-containing protein, CDC48	5.7 × 10^6^	67.91
Tb927.3.3330	Q57V53	HSP20	Heat shock protein 20	5.2 × 10^6^	4.52

The proteins are listed as described for Table 1

**TABLE 4:** Most enriched proteins co-precipitated with the eYFP-tagged EIF4G3 in *Trypanosoma brucei* procyclic cells.

TriTrypDB Accession	Protein ID	Name	Description	Protein Intensity	Ratio
**Bait**					
Tb927.8.4820	Q57UX0	EIF4G3	Eukaryotic translation initiation factor 4 gamma 3	6.6 × 10^7^	57.11
**Translation initiation factors**					
Tb927.6.1870	Q585M4	EIF4E4	Eukaryotic translation initiation factor 4E-4	9.6 × 10^7^	17.15
Tb927.9.9290	Q38E63	PABP1	Polyadenylate-binding protein 1	5.9 × 10^7^	8.32
Tb927.11.11590	Q383C1	EIF3E	Eukaryotic translation initiation factor 3 subunit E	1.6 × 10^6^	∞
**Ribosomal Protein**					
Tb927.10.7340	Q38AM9	RPS24	40S ribosomal protein S24	3.1 × 10^7^	16.92
**RNA binding proteins**					
Tb927.6.2200	Q584T6	PARK7	DJ-1 family protein	5.9 × 10^6^	17.76
Tb927.10.11270	Q389L2	RBP23	RNA-binding protein 23	2.9 × 10^6^	8.7
**Intracellular transport and cell motility**					
Tb927.2.4580	Q586Q9	UNC119	UNC119	7.3 × 10^6^	124.9
Tb927.3.860	Q57WW9	ACP	Acyl carrier protein	4.8 × 10^6^	34.14
Tb927.11.13650	Q382H2	CYB5	Cytochrome b5	2.5 × 10^6^	28.45
Tb927.10.2190	Q38C25	DUFF667	DUFF667	1.2 × 10^6^	35.76
**Proteases and peptidases**					
Tb927.10.6030	Q38B02	PSA1	Proteasome subunit alpha type 1	5.6 × 10^6^	12.49
Tb927.3.3410	Q57V45	DNPEP	Aspartyl aminopeptidase	2.8 × 10^6^	18.95
Tb927.5.1930	Q57ZR2	SPCS	Signal peptidase subunit	2.1 × 10^6^	61.43
**Other enzymes**					
Tb927.7.4520	Q57X68	TXNL	Thioredoxin-like	1.7 × 10^7^	16.25
Tb927.11.8970	Q384D1	RPIA	Ribose 5-phosphate isomerase	1.7 × 10^7^	21.98
Tb927.7.4480	Q57X64	HNT1	Adenosine 5-monophosphoramidase	7.5 × 10^6^	20.16
Tb927.10.5760	Q38B28	ADKF	Adenylate kinase	5.4 × 10^6^	9.74
Tb927.5.1710	Q57ZP0	P18	ATP synthase F1 subunit p18, mitochondrial	4.8 × 10^6^	∞
Tb927.7.840	Q57VT0	ATP5M	Mitochondrial ATP synthase subunit	2.6 × 10^6^	11.26
Tb927.8.5600	Q57X22	TALDO	Transaldolase	2.6 × 10^6^	37.99
Tb927.10.6950	Q4FKJ2	SMT	Sterol 24-c-methyltransferase	2 × 10^6^	18.04
Tb927.7.2710	Q57Y81	CYB5R	NADH-cytochrome b5 reductase	1.8 × 10^6^	14.25
Tb927.8.690	Q57YG1	PIN1	Peptidyl-prolyl cis-trans isomerase	1.4 × 10^6^	8.11
Tb927.7.1300	Q57WS0	PDI	Protein disulfide isomerase	1.2 × 10^6^	124.48
**Uncharacterized Protein**					
Tb927.7.4530	Q57X69	UCP4530	Uncharacterized protein, conserved	6.4 × 10^6^	25.47

The proteins are listed as described for Table 1, but with enrichment ratios >8 and intensities >10^6^.

A different pattern emerges for the EIF4E4/EIF4G3 complex (Figure 2, Tables 3, 4), with four proteins found among those most enriched for these two subunits but which are not enriched with either EIF4E3 or EIF4G4. These include both the EIF4E4 and EIF4G3 subunits as well as PABP1 and RBP23, an RNA-binding protein first identified as enriched with *Leishmania* and *T. brucei* PABP1 (de Melo Neto et al., 2018; Zoltner et al., 2018) and subsequently found to bind directely to PABP1 *in vitro* (Assis et al., 2021). One other protein found among those most enriched with EIF4E4 was also found enriched with EIF4G3 only, though with a lower enrichment than required for inclusion in Figure 2: ERP1, one of eight Emp24-related proteins found in *T. brucei*, proteins known to interact with the cytosolic coat protein (COP) complexes and with roles associated with endoplasmic reticulum mediated export (Kruzel et al., 2017). Despite a larger set of proteins enriched with EIF4E4, in comparison with EIF4E3, a smaller proportion of those, or ∼35% (18/51), were not found enriched >2-fold with any of the other tagged proteins (Supplementary Table S2). In fact, roughly 60% of the proteins enriched with EIF4E4 were also enriched with its main partner EIF4G3 (31/51), with one-third also enriched with EIF4G4 (17/51). In contrast, no proteins are enriched with EIF4E4 and EIF4E3 only. The topmost protein by far specifically associated with EIF4E4 (>65-fold enrichment) was the CDC48/VCP (valosin-containing protein), an eukaryotic/archaean ATPase chaperone with roles in protein and organelle degradation, cell-cycle regulation and cell signaling (Barthelme and Sauer, 2016). Regarding EIF4G3, roughly 70% of the enriched proteins were not found enriched with the remaining three proteins investigated here (Supplementary Table S2). The topmost EIF4G3-specific proteins include UNC119, known to participate in the intraflagellar transport of lipidated proteins (Pandey et al., 2020), the uncharacterized Tb927.7.4530, and a homologue of the human PARK7 (DnaJ-1) chaperone. A large number of enzymes were also seen substantially enriched mainly with EIF4G3, but sometimes also with EIF4G4 or the two eIF4E homologues, although any functional relevance of their co-precipitation with the tagged proteins remains to be determined. Overall, the profile of proteins co-precipitating with the eYFP-tagged baits confirms the conservation and integrity of the EIF4E3/EIF4G4 and EIF4E4/EIF4G3 complexes and the specific association of EIF4E4/EIF4G3 with PABP1, RBP23 and possibly EIF4AI.

### Functional assessment and pair-wise comparison of proteins co-precipitating with the EIF4E4/EIF4G3 and EIF4E3/EIF4G4 complexes

**FIGURE 3:**
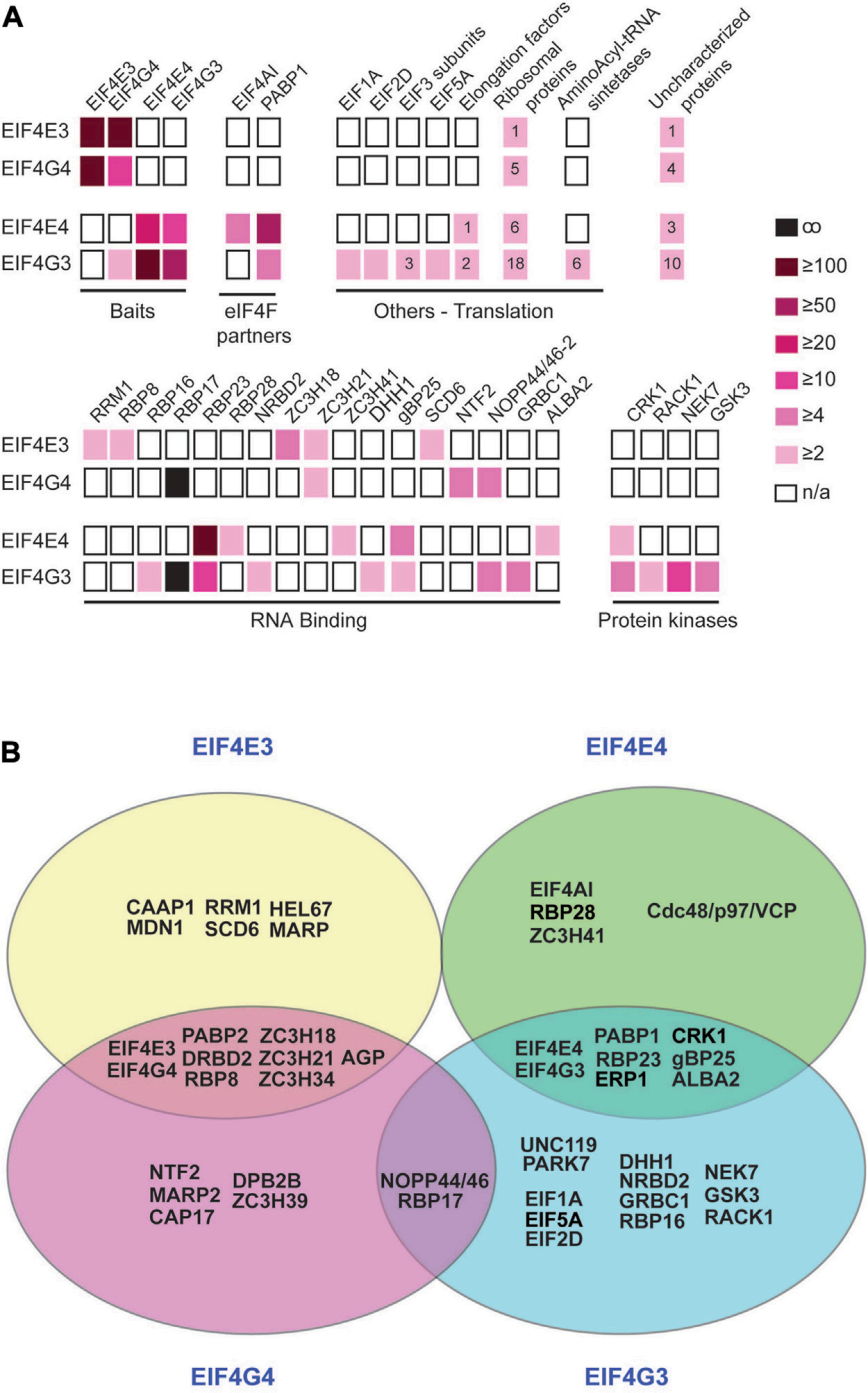
Comparative overview of selected proteins co-precipitated with the EIF4E3/EIF4G4 and EIF4E4/EIF4G3 complexes. **(A)** Proteins co-immunoprecipitated with each of the two sets of eIF4E and eIF4G homologues and belonging to selected functional categories are represented by boxes coloured according to the enrichment ratios derived from Supplementary Table S2. The numbers inside the boxes indicate the number of subunits of the named complex (including ribosomes) that have equivalent enrichment ratios. **(B)** Venn diagram summarizing the major results derived from the analyses shown in **(A)** and in the Supplementary Figure S2. Proteins co-precipitated with individual subunits or with the eIF4E or eIF4G pairs are also represented. Most enzymes and hypothetical proteins we opted to exclude since their functional roles in translation and/or mRNA metabolism, if any, remain to be defined. For clarity and simplification, ribosomal proteins and other factors known to be required for translation per se were also not included, as well as many of the proteins found to be exclusively co-precipitated with EIF4G3.

For a clearer understanding of functional roles and possible distinctions between the two complexes, we compared the larger set of proteins co-precipitated with EIF4E3/EIF4G4 and EIF4E4/EIF4G3 using the >2-fold enrichment criteria (Supplementary Table S2). Selected proteins were grouped according to known functional features and possible links to eIF4F function (summarized in Figure 3A). Although the EIF4E3, EIF4E4 and EIF4G4 baits only co-precipitated the corresponding eIF4E/eIF4G partners, EIF4G3 also pulled down EIF4G4. EIF4AI, the third subunit of eIF4F, was clearly associated with EIF4E4, with no enrichment seen with the other EIF4E3/EIF4G4 complex, while PABP1 was the only PABP homologue specifically enriched with the EIF4E4/EIF4G3 pair in comparison with the negative control. When other translation initiation factors were investigated, several of those were seen enriched with EIF4G3, including EIF1A and eIF3 subunits (Figure 3A). EIF4G3 was also seen to co-precipitate many other proteins directly involved with translation, including elongation factors, aminoacyl-tRNA synthetases and a larger number of ribosomal proteins. Several RNA-binding proteins were also seen to be differentially enriched with the different tagged proteins, including some with a clear association with subunits belonging to one of the two complexes. As described above, RBP23 was clearly associated with both EIF4E4 and EIF4G3, but other RNA-binding proteins (RBP16, RBP28, NRBD2, gBP25, GRBC1 and ALBA2), the zinc-finger protein ZC3H41 and the RNA helicase DHH1 were also found with either of these subunits, albeit with more reduced enrichments. In contrast, RRM1, RBP8, SCD6, NTF2 and the zinc-fingers ZC3H18 and ZC3H21 were found enriched with either or both subunits of the EIF4E3/EIF4G4 complex, while both eIF4Gs were associated with RBP17 and NOPP44/46-2.

DRBD4, the protein used as negative control for the immunoprecipitations here, was chosen as a negative control due to its nuclear localization (Goos et al., 2017). However, DRBD4 is an mRNA-associated protein (Stern et al., 2009), so that any protein that binds to the mRNAs prior to their export to the cytoplasm, and co-precipitates with DRBD4, could be missed from our analyses. To extend our analysis of proteins specifically associated with the complexes, we compared pairwise the two sets of eIF4E and eIF4G homologues (Supplementary Table S3). These analyses revealed additional proteins enriched with both subunits of the EIF4E3/EIF4G4 complex in comparison with the EIF4E4/EIF4G3 pair (Supplementary Figure S2). These include a very large protein of unknown function and mainly consisting of a large number of 24 amino acids repeats (AGP; Tb927.4.2070), as well as PABP2 and RNA-binding proteins such as DRBD2 and the zinc-finger protein ZC3H34. There are also enrichments of the DEAD-Box helicases HEL67 and DBP2B with EIF4E3 and EIF4G4, respectively. In this analysis, no additional proteins were identified as being associated with EIF4E4/EIF4G3. Figure 3B summarizes selected proteins that might be of interest in further studies and which are most likely to have specific roles associated with the two eIF4F-like complexes investigated, based on the analyses reported here. This selection is validated by the presence with EIF4E4/EIF4G3 of EIF4AI, PABP1, RBP23 and CRK1, proteins previously known to be associated with this complex from work with *T. brucei* and/or *Leishmania* (Moura et al., 2015; An et al., 2018; de Melo Neto et al., 2018; Zoltner et al., 2018; Assis et al., 2021). It is also reinforced by the specific co-precipitation with the EIF4E3/EIF4G4 pair of proteins previously found with the *T. brucei* PABP2, such as DRBD2 and ZC3H39 (Zoltner et al., 2018).

### Steady state levels of EIF4E3 and EIF4G4 are necessary for maintenance of cell morphology

**FIGURE 4:**
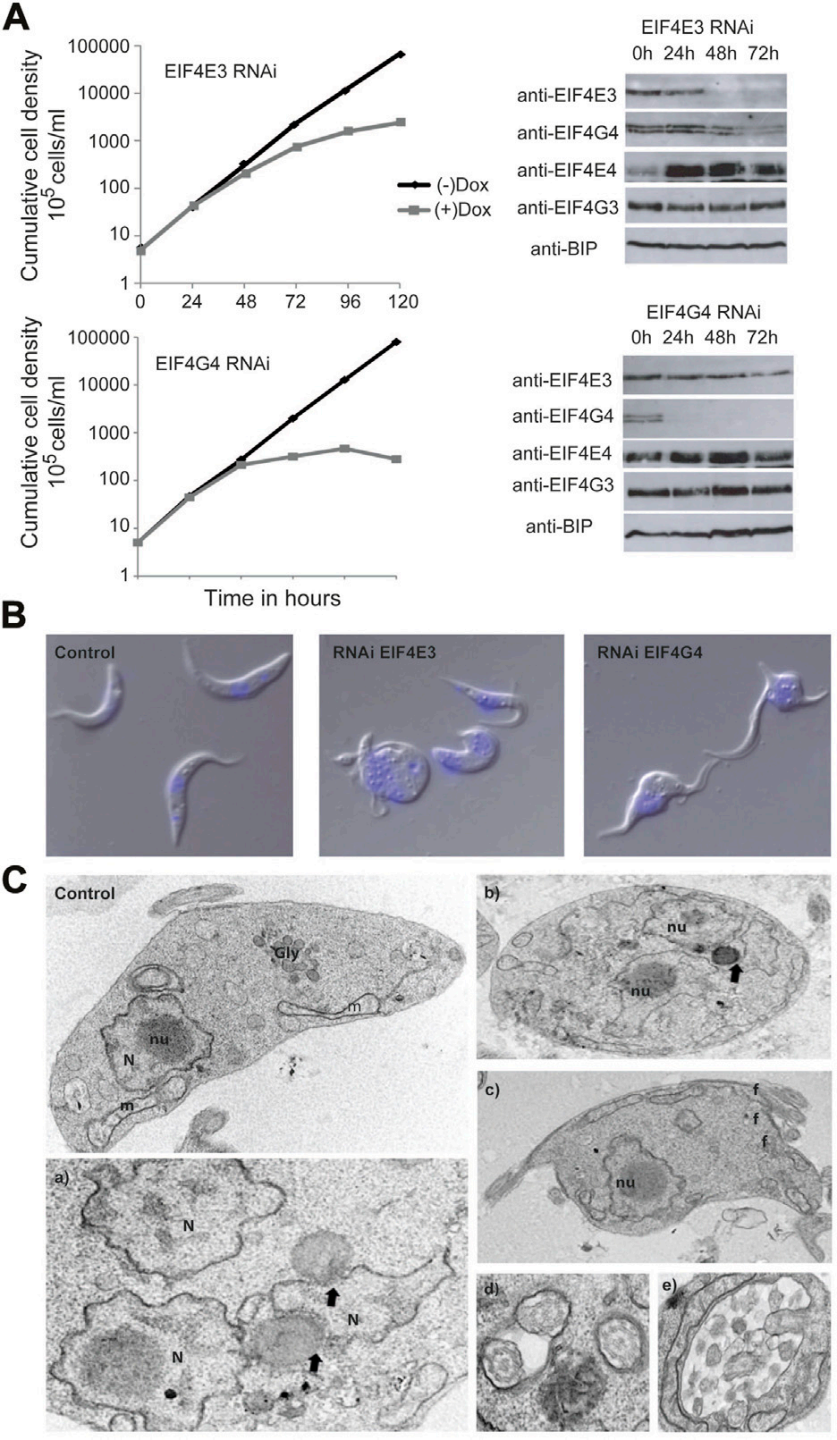
Analysis of the EIF4G4 depletion effects in cell morphology **(A)** Representative growth curves of *T. brucei* procyclic cells submitted to the EIF4E3 and EIF4G4 RNAi after 24, 48, and 72 h Doxycycline induction (+Dox) and without RNAi induction (-Dox). Western blots using the polyclonal antibodies anti-EIF4E3, anti-EIF4E4, anti-EIF4G4 and anti-EIF4G3 in the RNAi induced cell lines. Anti-BIP was used as a loading control. **(B)** Confocal microscopy of *T. brucei* procyclic cells with the EIF4E3 RNAi after 48 h of induction with Doxycycline. Cells with an aberrant phenotype consisting of multiple nuclei and flagella can be observed in the RNAi samples. In blue, DNA stained with DAPI. **(C)** Electron microscopy of T. brucei procyclic cells after 48 h of EIF4G4 RNAi induction. Control—T. brucei procyclic cell without RNAi induction, showing the preserved organelles: nucleus (N) and nucleolus (nu), glycosome (Gly) and mitochondrion (m). a) and b) Cells displaying multiple nuclei (N) and disintegration of the nucleolus. Black arrows pointing membrane bridges between nuclear envelope membrane and lipid-like inclusions. c) Cell exhibiting multiple flagella (f). d) Multiple flagella in the same flagellar pocket. e) Atypical exocytic activity in the flagellar pocket.

RNA interference-mediated depletion of both EIF4G3 and EIF4G4 in *T. brucei* procyclic cells has been previously shown to lead to loss of cell viability and, for EIF4G3, inhibition of translation (Moura et al., 2015). In contrast, depletion of EIF4E3 and EIF4E4 led to markedly different outcomes, with knock down of EIF4E3 resulting in a loss of cell viability in both procyclic and bloodstream cells, while EIF4E4 depletion in procyclic cells did not cause any major effects in cell growth, although the viability in bloodstream forms is impaired (Freire et al., 2011). The induction of EIF4G4 RNAi also caused a distinct phenotype characterized by aberrant cells with round shape and multiple nuclei and flagella (Moura et al., 2015). A similar phenotype was more recently described after the single knockout of EIF4E3 in *Leishmania* (Shrivastava et al., 2019b). The similarity between the morphological alterations seen after RNAi of EIF4E3 and its EIF4G4 partner led us to further characterize the phenotype here. First, we performed parallel RNAi experiments to evaluate the effect of depletion of EIF4E3 and EIF4G4, and how this affected the expression of the EIF4E4/EIF4G3 complex. As expected, both knockdowns led to loss of viability with a somewhat stronger phenotype seen following the EIF4G4 RNAi (Figure 4A). When the expression of individual subunits was assessed by western blot analysis, knockdown of EIF4E3 led to a reduction of its levels starting 24 h after RNAi induction but substantially more effective at the 48 h time point. Depletion of EIF4E3 was accompanied by a substantial reduction in EIF4G4 levels, noticeable 48 and 72 h after the start of RNAi. In contrast, depletion of EIF4E3 led to an increase in the levels of EIF4E4, despite no equivalent changes seen for its partner EIF4G3. When the EIF4G4 RNAi was evaluated, depletion of EIF4G4 was very significant even at the 24 h time point but it was not accompanied by any substantial loss of EIF4E3, nor major changes in the EIF4E4/EIF4G3 levels. These results indicate a tight link between the levels of EIF4G4 with those of EIF4E3, as seen after EIF4E3 depletion. However, EIF4E3 levels were unaffected by EIF4G4 depletion, perhaps due to the levels of EIF4E3 being 10-fold higher than those of EIF4G4 (Freire et al., 2011; Moura et al., 2015).

We also monitored changes in morphology induced by the two sets of RNAi knockdowns. First, (Figure 4B), cells with similar morphological alterations were seen using light microscopy for both EIF4E3 and EIF4G4 knockdowns, albeit the incidence of affected cells was different (Supplementary Figure S4). For EIF4E3 silencing, 5%–9% of cells were aberrant (with a round shape and/or having multiple flagella) 48 h after RNAi induction, increasing to ∼25% at 72 h and 30%–35% aberrant cells at 120 h. In contrast, for EIF4G4, ∼45% of the cells had an aberrant phenotype 48 h after RNAi induction, with over 60% at the 120 h time point. While EIF4E3 depletion induces a more limited effect than EIF4G4, it is noteworthy that the aberrant phenotype for the cells with the EIF4E3 RNAi coincides not only with a decrease in EIF4E3 abundance but also with a decrease in the levels of EIF4G4, as seen with the expression analysis. This observation raises the possibility that the changes in morphology seen after the EIF4E3 RNAi may be related to the reduction in EIF4G4 levels.

Ultrastructural analysis by transmission electron microscopy of cells after EIF4G4 RNAi indicated aberrant division, suggestive of defective cytokinesis, with many cells having multiple nuclei and flagella (Figure 4C, panels a, b, c and d). However, glycosomes and acidocalcisomes were not significantly affected. Interestingly, cytoplasmic inclusions, resembling lipid droplets, were observed in the vicinity of nuclei and, less frequently, near the mitochondria. Ribosomes were seen in association with these inclusions, and membrane bridges between nuclear envelope membranes and lipid-like inclusions were also found. In some cells, a single lipid droplet was seen simultaneously contacting two nuclei (Figure 4C, panel e). Vesicles of varying size were also seen within the flagellar pocket. In addition, mitochondria with altered morphologies were observed in the EIF4G4 RNAi induced cells, when compared with non-induced ones. Overall, these morphological changes suggest a major impact on multiple processes related to cell division and cellular homeostasis induced by EIF4G4 depletion.

### mRNA populations differentially co-precipitated with EIF4E3 and EIF4E4

**FIGURE 5:**
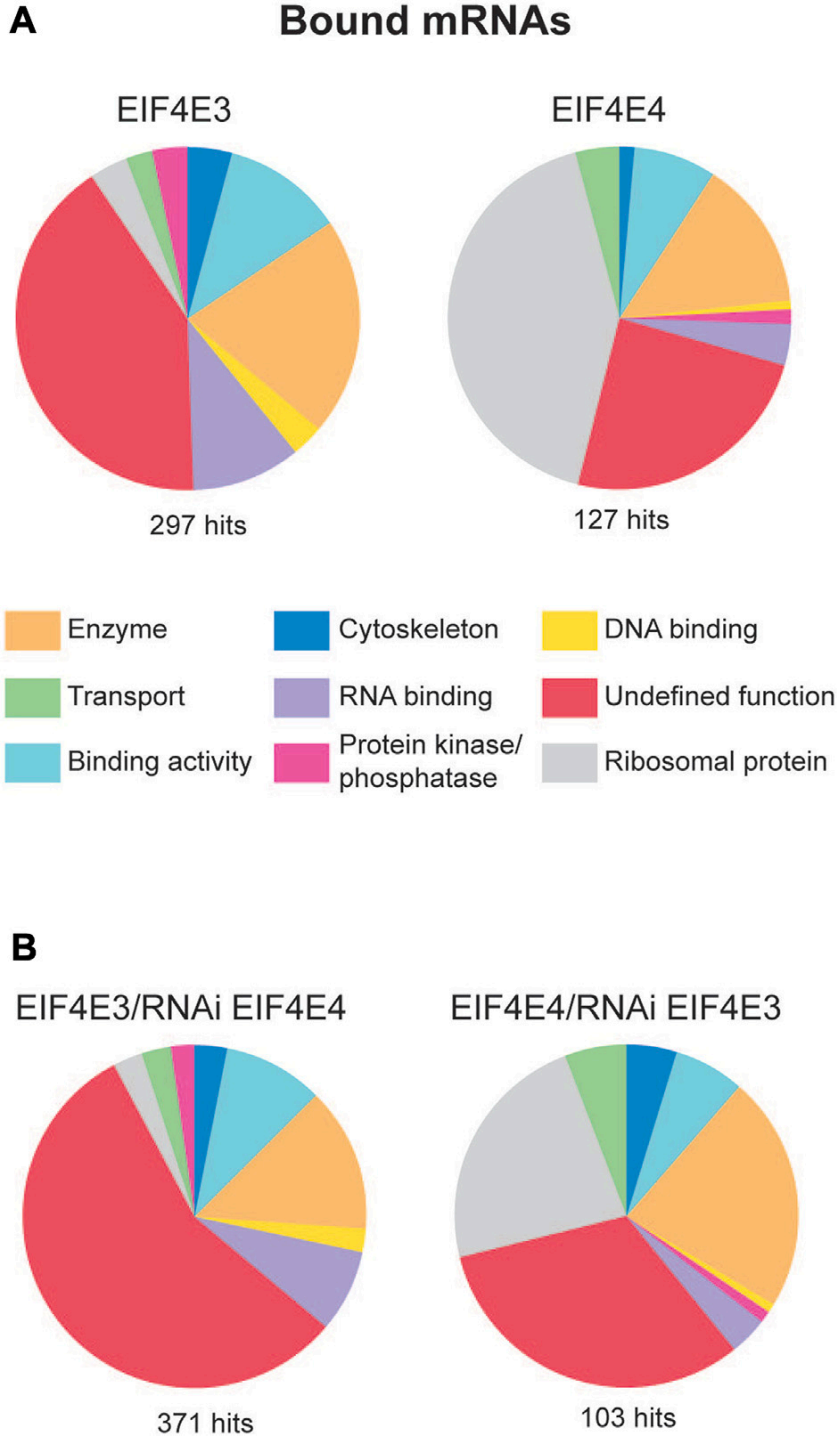
mRNA populations associated with EIF4E3 and EIF4E4. **(A)** mRNA groups associated with EIF4E3 and EIF4E4 from *T. brucei* (Illumina sequencing). Enriched mRNAs were manually classified and grouped using the gene ontology (GO) terms according to their molecular function. “Enriched” means at least 2-fold more abundant than in the negative control. **(B)** mRNA groups associated with EIF4E3 after EIF4E4 RNAi, and with EIF4E4 after EIF4E3 RNAi, manually classified using the same parameters as figure **(B)**.

To determine whether the EIF4E3/EIF4G4 and EIF4E4/EIF4G3 complexes recruit the same or different sets of mRNAs, we focused on the two eIF4E subunits as these bind mRNAs through 5’ capped ends. CLIP (cross-link-IP)-seq experiments were set up using cellular extracts from *T. brucei* procyclic cells exposed to UV-crosslinking and lysed through nitrogen cavitation. For the IPs we used the previously described affinity-purified antibodies directed against native EIF4E3 and EIF4E4 (Freire et al., 2011) coupled to magnetic beads. For reasons detailed further below, we also performed these experiments with extracts from cell lines generated with the RNAi plasmids (IPs for EIF4E3 using cells transfected with the EIF4E4 RNAi plasmid and IPs for EIF4E4 using cells with the EIF4E3 construct). Immunoprecipitated samples were submitted to RNA extraction, cDNA synthesis and sequencing using the Illumina technology. First, to assess the quality of the sequencing and its reproducibility, we examined the mRNAs identified with the negative controls, lacking antibodies (Supplementary Table S4). The most abundant mRNAs here included a large number of ribosomal protein mRNAs as well as transcripts encoding tubulin subunits, histones, translation elongation factors, the procyclin surface antigens and others, all generally compatible with previously published transcriptome data of *T. brucei* procyclic cells (Kolev et al., 2010; Siegel et al., 2010; Archer et al., 2011; Naguleswaran et al., 2018), and representing highly abundant mRNAs with non-specific RNA binding to the beads. To identify the transcripts co-precipitated with EIF4E3 and EIF4E4, these were filtered by selecting those with a greater than twofold average enrichment, relative to the average from the corresponding negative controls. The analyses of the enriched mRNAs identified different patterns of mRNAs targeted by each eIF4E homologue, with a higher number of transcripts associated with EIF4E3 (297) than with EIF4E4 (127) (Figure 5A, Supplementary Table S5).

Next, the enriched transcripts were grouped according to molecular functions, defined using Gene Ontology (GO) terms but modified by us in order to improve clarity (Figure 5A). In particular, the “Undefined Function” groups transcripts encoding both hypothetical or unknown proteins and others whose function could not be classified within the defined categories. In the case of EIF4E3, this constituted the main category, representing nearly half of all enriched transcripts, with the remainder divided into “Enzyme”, “RNA-Binding” and “Binding Activity” categories. Within the “Enzyme” category, nine mRNAs encoded enzymes with protein kinase or protein phosphatase functions. Notably, approximately 40% of EIF4E4-bound transcripts encode ribosomal proteins, whereas these constituted less than 4% of the EIF4E3-bound transcripts. EIF4E4-bound mRNAs also fell into the “Binding Activity” and “Enzyme” categories, but only one transcript encoding a “Protein Kinase” was found with EIF4E4 (Tb927.9.14430). A more detailed analysis of the most enriched mRNAs bound to each factor reinforces the functional distinctions of their encoded proteins. Several highly expressed mRNAs were clearly co-precipitated with EIF4E3 only, such as those encoding histones, surface antigens (GPEET procyclin, BARP), cytoskeletal proteins (dynein, kinesin) and a number of RNA-binding proteins. Table 5 lists the 30 most enriched mRNAs found with each eIF4E homologue, EIF4E4 co-precipitates with 15 mRNAs encoding ribosomal protein subunits, transcripts which are absent from the equivalent EIF4E3 list. Notably, many of the transcripts most enriched with EIF4E3 encode RNA-binding proteins.

**TABLE 5:** List of the 30 most enriched mRNAs co-precipitated with EIF4E3 and EIF4E4 from *Trypanosoma brucei* procyclic cells.

EIF4E3			EIF4E4		
TriTrypDB Accession	Description	Ratio	TriTrypDB Accession	Description	Ratio
Tb927.6.510	GPEET procyclin	5.14	Tb927.10.13500	60S ribosomal protein L10a	2.66
Tb927.11.510	RNA-binding protein, UBP2	4.58	Tb927.11.11830	40S ribosomal protein S17	2.47
Tb927.6.3490	Zinc finger protein 1	4.31	Tb927.11.6300	40S ribosomal protein S5	2.38
Tb927.10.1480	Hypothetical protein	4.30	Tb927.8.3110	Mitochondrial ribosomal protein S9	2.27
Tb927.6.640	ApaH-like phosphatase ALPH1	4.17	Tb927.8.6890	Succinate dehydrogenase subunit	2.25
Tb927.1.2310	Hypothetical protein	3.94	Tb927.10.3840	60S ribosomal protein L18a	2.22
Tb927.7.3970	Hypothetical protein	3.56	Tb927.11.6180	60S ribosomal protein L28	2.15
Tb927.5.2260	Conserved protein	3.50	Tb927.4.3880	Receptor-type adenylate cyclase GRESAG 4	2.14
Tb927.2.3880	Heterogeneous RNP F/H homologue	3.24	Tb927.8.4630	Hypothetical protein	2.11
Tb927.11.500	RNA-binding protein, UBP1	3.22	Tb927.9.15380	NADH-ubiquinone oxidoreductase subunit	2.08
Tb927.10.12730	Hypothetical protein	3.02	Tb927.11.9710	60S ribosomal protein L10a	2.03
Tb927.5.820	Hypothetical protein	2.99	Tb927.10.14740	Hypothetical protein	2.02
Tb927.6.5020	Cyclin 7, putative	2.97	Tb927.11.740	EIF5A	1.97
Tb927.8.870	CAMK/CAMKL protein kinase	2.82	Tb927.3.1370	40S ribosomal protein S25	1.87
Tb927.6.4280	Glyceraldehyde 3-phosphate dehydrogenase, glycosomal	2.77	Tb927.1.710	Phosphoglycerate kinase	1.84
Tb927.2.2770	Hypothetical protein	2.75	Tb927.10.3850	Hypothetical protein	1.82
Tb927.11.3760	Hypothetical protein	2.66	Tb927.11.3590	40S ribosomal protein S4	1.82
Tb927.5.2160	Conserved protein	2.56	Tb927.9.8420	QM-like protein	1.79
Tb927.10.3970	Hypothetical protein	2.51	Tb927.9.7620	60S ribosomal protein L11	1.74
Tb927.3.2960	Inosine-adenosine-guanosine-nucleosidehydrolase	2.44	Tb927.11.12040	Cytochrome c oxidase component	1.73
Tb927.6.5010	Hypothetical protein	2.40	Tb927.1.3180	40S ribosomal protein S11	1.71
Tb11.v5.0722	Variant surface glycoprotein (VSG)-related, putative	2.38	Tb927.10.5360	40S ribosomal protein S10	1.69
Tb927.5.800	Casein kinase I, isoform 2	2.38	Tb927.10.3280	60S ribosomal proteins L38	1.68
Tb927.8.5440	Flagellar calcium-binding 24 kDa protein	2.32	Tb927.9.8100	Nascent polypeptide associated complex subunit	1.66
Tb927.11.16820	Hypothetical protein	2.32	Tb927.10.3830	Hypothetical protein	1.66
Tb927.3.4710	Flagellum attachment zone protein	2.29	Tb927.2.6090	60S ribosomal protein L44	1.65
Tb927.10.15310	Hypothetical protein	2.28	Tb927.9.15420	60S ribosomal protein L32	1.65
Tb927.7.2660	Zinc finger protein, ZC3H20	2.28	Tb927.3.4500	Fumarate hydratase class I	1.64
Tb927.5.1990	Hypothetical protein	2.25	Tb927.8.3530	Glycerol-3-phosphate dehydrogenase, glycosomal	1.64
Tb927.11.3310	Ubiquitin-conjugating enzyme	2.24	Tb927.7.2980	Nitroreductase family	1.61

The ratio column represents the log_2_ enrichment ratios in comparison with the negative control.

As an independent verification for the RNA co-precipitation experiments, we carried out another set of IPs using the same antibodies but with extracts prepared from cells without prior UV exposure. For these experiments the cells were lysed with Nonidet NP-40 detergent and the IPs were carried out using protein-A Sepharose beads, again with beads only used as negative controls. Sequencing was performed using the Solid platform and qualitative differences evaluated after grouping with functional terms as described above (Supplementary Figure S4 and Supplementary Table S6). EIF4E3 and EIF4E4 had similar numbers of enriched mRNAs (223 and 225), with overall results confirming those seen in the CLIP experiment. These reinforce the distinct sets of mRNAs bound by each of the complexes studied here, with the most notable result being the specific association of the EIF4E4/EIF4G3 complex with mRNAs encoding ribosomal proteins, a result supported by the specific association recently found between the *Leishmania* PABP1 and RBP23 with the same set of mRNAs (Assis et al., 2021).

### Effect of RNAi mediated depletion on proteins and mRNAs co-precipitated with EIF4E3 and EIF4E4

The lack of altered phenotype resulting from EIF4E4 RNAi in procyclic cells (Freire et al., 2011), contrasted with the strong phenotype observed for its EIF4G3 partner (Moura et al., 2015). One possible explanation would be redundancy between the EIF4E3 and EIF4E4 homologues as EIF4G3 partners. Alternatively, the EIF4E3/EIF4G4 complex may recruit mRNAs that would be otherwise bound by EIF4E4/EIF4G3, although the differences in RNAi phenotype between EIF4E4 and EIF4G3 would remain unexplained. To investigate these possibilities, we used CLIP-seq to assess the mRNAs associated with EIF4E3 in cells submitted to RNAi-mediated EIF4E4 knockdown (48 h timepoint), with reciprocal experiments done to investigate any changes in EIF4E4-bound mRNAs after EIF4E3 depletion. When individual transcripts are compared, approximately 90% of the 30 topmost EIF4E3- or EIF4E4-bound transcripts remained in the corresponding IPs carried out after RNAi (Figure 5B and Supplementary Table S7). For EIF4E4-bound mRNAs, however, there was a reduction in the number of bound ribosomal protein transcripts after EIF4E3 RNAi. While no transcripts could be found shifting from EIF4E4 to EIF4E3 upon EIF4E4 RNAi, a number of mRNAs previously only found with EIF4E3 co-precipitated with EIF4E4 after EIF4E3 depletion, although these do not seem to have any relevant features in common.

Assays were also set up to identify any changes in EIF4E3 protein partners after EIF4E4 depletion. Using the native anti-EIF4E3 antibodies, IP reactions were setup as described for the CLIP-Seq experiments before and after induction of EIF4E4 RNAi, with the samples sent for mass spectrometry identification of co-precipitated proteins. Except for a minor increase in the presence of PABP1 with EIF4E3 (∼1.7 fold), the results did not show any significant differences, before and after EIF4E4 depletion, for proteins associated with EIF4E3 which are known to be functionally relevant (Supplementary Table S8). These observations therefore indicate that EIF4E3 is not able to compensate for the lack of EIF4E4 in mediating translation of the EIF4E4-bound mRNAs.

## Discussion

The large number of eIF4E and eIF4G paralogues identified in trypanosomatids implies extensive roles in regulating translation. While details remain unclear, the simultaneous and constitutive expression of many paralogues in both *Leishmania* and *Trypanosoma brucei* (Dhalia et al., 2005; Freire et al., 2011; Pereira et al., 2013; Tinti and Ferguson, 2022) suggests limited involvement in stage-specific functions. The greater abundance of EIF4E3 and EIF4E4, considering the original quantitation of the EIF4E1, EIF4E2, EIF4E3 and EIF4E4 paralogues (Dhalia et al., 2005; Freire et al., 2011), suggested more prominent roles for these proteins and associated complexes. EIF4E5 and EIF4E6, however, were not included in those studies which differ from a recent, high throughput, analysis suggesting that, except for EIF4E2, all *T. brucei* eIF4Es and eIF4Gs have similar abundances (Tinti and Ferguson, 2022). Moreover, neither EIF4E1 nor EIF4E2 seem able to form eIF4F-like complexes (Zinoviev et al., 2011; Freire et al., 2018; Terrao et al., 2018) and roles in translation for these paralogues would thus involve novel aspects yet to be defined. EIF4E1 in *T. brucei* functions as a translation inhibitor (Terrao et al., 2018; Falk et al., 2021), but contradictory evidence has been reported from the *Leishmania* orthologue (Zinoviev et al., 2011; Meleppattu et al., 2015). For those eIF4E and eIF4G homologues found forming eIF4F-like complexes, with five of those identified so far (Freire et al., 2017), a possibility would be for the different complexes to be associated with distinct sets of mRNA targets, possibly also involving the action of distinct protein partners. Indeed, this seem to be the case for the EIF4E6/EIF4G5 based complex, as recently reported (Melo do Nascimento et al., 2021).

**FIGURE 6:**
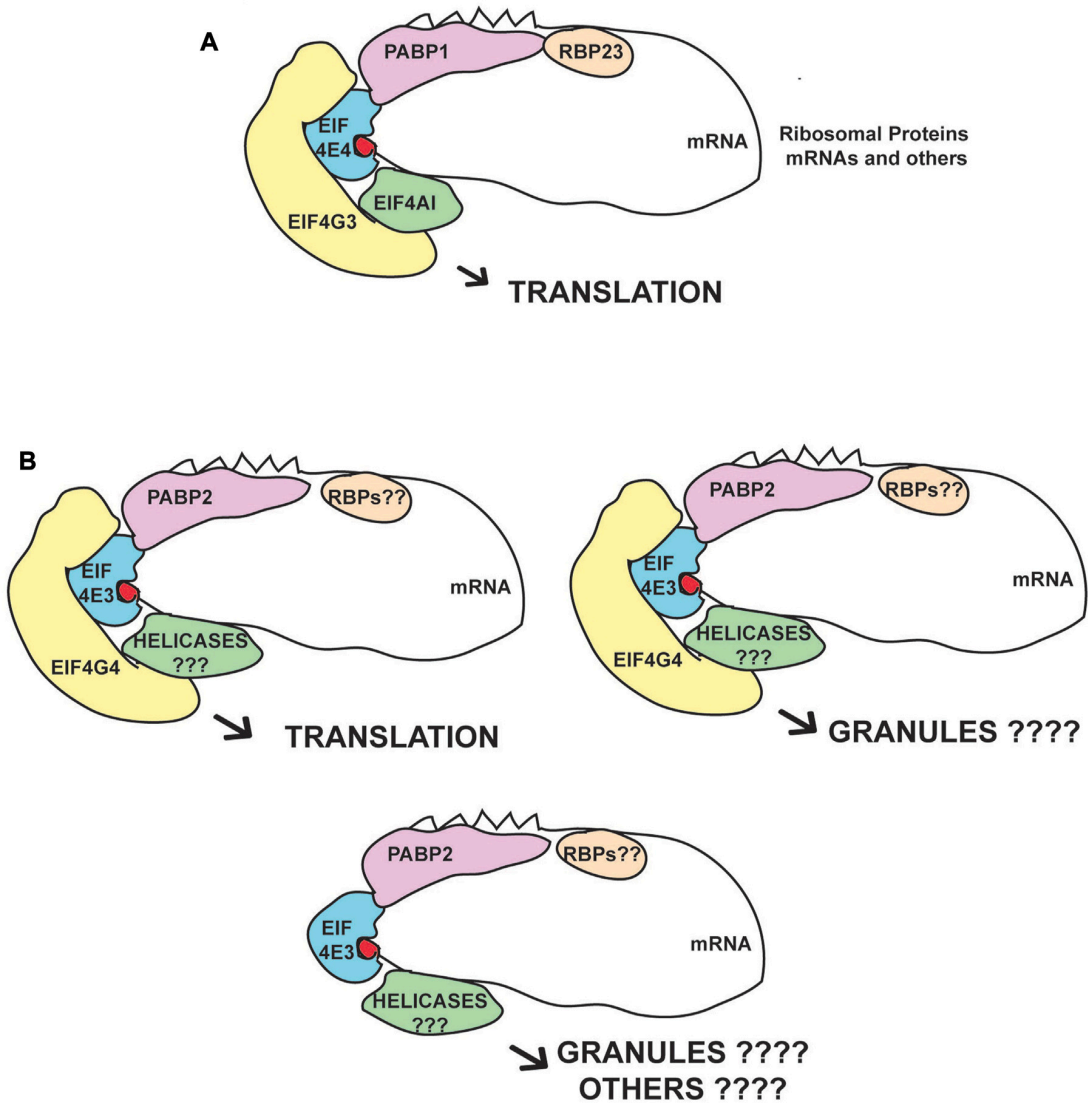
Proposed eIF4F-like complexes and variations based on the EIF4E3/EIF4G4 and EIF4E4/EIF4G3 subunits. **(A)** Consensus complex based on the immunoprecipitation data and on the known interactions between the EIF4E4/EIF4G3 subunits and its confirmed PABP1, EIF4AI and RBP23 partners. This complex seems to act mainly on translation with the mRNAs encoding ribosomal proteins as their major targets. **(B)** Complexes based on the EIF4E3/EIF4G4 subunits and their association with PABP2. Roles in both translation and other processes associated with RNA granules are postulated. EIF4E3 may also function in the absence of EIF4G4, due to the large difference in abundance between the two proteins, but the pairing between EIF4G4 and EIF4E3 is likely essential for translation and viability. We propose that the different RNA helicases and RBPs that specifically associate with these complexes modify their functions.

Our new analysis, performed after cryogrinding and without detergent, not only confirmed previous results from *Leishmania* (Zinoviev et al., 2011, 2012b; Shrivastava et al., 2019a), but also identified additional proteins enriched with EIF4E4/EIF4G3 and EIF4E3/EIF4G4. A similar large cohort of proteins was identified using this protocol focusing on mRNA maturation factors, validating the approach as capable of increasing representation of mRNA processing interactions (Inoue et al., 2022). Although at this stage we cannot completely rule out some impaired functionality caused by the eYFP tagging of the eIF4G homologues only, since we were unable to generate cell lines lacking the untagged eIF4Gs, the efficient tagging observed for the first allele despite the likely haplo-insufficiency observed after the gene deletion attempts, the mass-spectrometry data compatible with translation factors and the quite distinct association with protein partners and mRNA targets are consistent with both tagged eIF4G homologues being mostly, if not fully, functional. The large number of proteins found in common with both EIF4G3 and EIF4G4, and to a minor extent with EIF4E4, is expected, due to their related roles in translation, while the differences seen for EIF4E3 may reflect the fact that it may have other functions beyond being a part of the EIF4E3/EIF4G4 complex. The present study then clearly defines major partners for the EIF4E4/EIF4G3 complex, while revealing a more diverse pattern of proteins associated with EIF4E3/EIF4G4. Proposed models for the different complexes based on the present and other work are shown in Figure 6.

A specific association between the EIF4E4/EIF4G3 complex and PABP1 was identified in *Leishmania* (da Costa Lima et al., 2010) and corroborated by studies investigating PABP-interacting partners in both *Leishmania* and *T. brucei* (Kramer et al., 2013; de Melo Neto et al., 2018; Zoltner et al., 2018). An association with RBP23 was also identified in some of these studies and by our more recent results confirming direct interaction between PABP1 and RBP23, and the specific co-precipitation of the EIF4E4/EIF4G3 complex with a tagged RBP23 (Assis et al., 2021). Likewise, a specific association with *Leishmania* EIF4AI has also been implied by a stronger interaction with EIF4G3, as compared with EIF4G4, and by mutagenesis studies where EIF4G3, but not EIF4G4, is impacted by mutations targeting the LNK motif, required for the EIF4AI interaction and localized within the proteins’ MIF4G domain (Moura et al., 2015). Indeed, the evidence implies that the complex formed by EIF4G3/EIF4E4/EIF4AI most closely resembles the canonical eIF4F complex described from metazoans, despite a novel interaction between EIF4E4 and PABP1 (Zinoviev et al., 2011; de Melo Neto et al., 2018; Dos Santos Rodrigues et al., 2018). The fact that EIF4E4, EIF4G3, PABP1, EIF4AI and RBP23 all stimulate expression in the tethering assay and/or are directly implicated in protein synthesis (Dhalia et al., 2006; Freire et al., 2011; Erben et al., 2014; Moura et al., 2015; Lueong et al., 2016), while EIF4AI, EIF4E4, EIF4G3 and PABP1 are also associated with polysomes (Kramer et al., 2013; Klein et al., 2015), clearly defines a complex that is active during translation initiation.

The mass spectrometry data for the EIF4E3/EIF4G4 complex suggests a preference for PABP2 which has not been clearly defined before. The PABP2 association also with the negative control used here, the nuclear protein DRBD4, may be explained by PABP2 shuttling between the nucleus and cytoplasm (da Costa Lima et al., 2010; Kramer et al., 2013). Further relevant proteins related to this complex include SCD6 and DRBD2, both identified as translational repressors that localize to cellular granules (Cristodero et al., 2014; Lueong et al., 2016; Wippel et al., 2019). In contrast, PABP2 associates with polysomes and is required for protein synthesis (da Costa Lima et al., 2010; Kramer et al., 2013; Erben et al., 2014). Several zinc-finger proteins co-precipitated with EIF4E3/EIF4G4 are translational activators in tethering assays, including ZC3H18, ZC3H21 and ZC3H34 (Erben et al., 2014; Lueong et al., 2016). ZC3H21 was further shown to be a procyclic-specific regulator of translation which recruits the MKT1-PBP1 complex and binds a restricted number of mRNA targets (Liu et al., 2020). The uncharacterized protein Tb927.11.2250, identified as an activator in the tethering assay, was found to be associated with the mRNA-binding proteome (Erben et al., 2014; Lueong et al., 2016) and localizes to stress granules (Fritz et al., 2015). Both EIF4E3/EIF4G4 are also substantially enriched with an uncharacterized protein (AGP; Tb927.4.2070) which may be linked to the cytoskeleton. Overall, the mixed profile of proteins co-precipitated with EIF4E3/EIF4G4 suggests more diverse roles for this complex. Indeed, this is consistent with the evidence seen for *Leishmania* EIF4E3, which localizes to stress-induced cytoplasmic granules upon stress, an indication of possible roles associated with mRNA storage (Shrivastava et al., 2019a). A relevant observation is the reduction in EIF4G4 levels seen here upon depletion of EIF4E3, which is in agreement with recent data from *Leishmania* (Shrivastava et al., 2019b). Although this observation requires further investigation, it does suggest that EIF4G4 is mostly non-functional in the absence of its EIF4E3 partner and that EIF4E3 may have roles independent from EIF4G4.

The specific association of unique protein partners with individual eIF4F subunits may reflect alternative roles or possible mechanisms associated with specific mRNA targets. One such example is CAAP1, one of the topmost proteins associated with EIF4E3, previously implicated in flagellar biosynthesis (Pham et al., 2020). Intriguingly, both EIF4E3 and EIF4E4 co-precipitate specifically with AAA-ATPases (associated with various cellular activities). EIF4E3 co-precipitates with Midasin (MDN1), a mostly nucleolar protein with roles in assembly and export of the large ribosomal subunit (Kressler et al., 2012), while EIF4E4 is enriched with Cdc48/p97/VCP (valosin-containing protein), a major player in various cellular pathways normally associated with protein degradation (Ye et al., 2017). As for EIF4G3 and EIF4G4, the large number of proteins co-precipitating exclusively or mainly with these two eIF4G homologues are most likely associated with translation initiation events. The large number of proteins specifically enriched with EIF4G3 highlights relevant properties yet to be clearly defined but are compatible with the strong phenotype observed after RNAi (Moura et al., 2015). The more limited profile of proteins enriched specifically with EIF4G4, the most prominent being Tb927.10.2240, might indicate more restricted functions. Tb927.10.2240 has a nuclear transport factor domain and was first found to co-precipitate with components of the *T. brucei* exon junction complex (Bercovich et al., 2009). Its *T. cruzi* orthologue (TcNTF2L) has been characterized in more detail recently and found to associate specifically with various proteins involved with the mRNA export machinery (Inoue et al., 2022). Tb927.10.2240 was also identified as a possible DRBD3 partner (Fernandez-Moya et al., 2012), as an activator in the tethering assay and part of the *T. brucei* mRNA-associated proteome (Erben et al., 2014; Lueong et al., 2016), but the relevance of its co-precipitation with EIF4G4 is unclear. In contrast to the lack of a clear association with EIF4AI, as seen for both EIF4E4/EIF4G3 subunits, other abundant DEAD-box helicases are specifically enriched with EIF4E3 and EIF4G4, including HEL67 (Tb927.10.14550) and DBP2B (Tb927.8.1510). Interestingly, HEL67 is closely related to human DDX3 and yeast Ded1, proteins found to be directly involved in translation initiation (Mokdadi et al., 2021). In *Leishmania*, the HEL67 orthologue has been implicated in the response to stress (Padmanabhan et al., 2016) and in both *T. brucei* and *Leishmania* it has been also seen to be involved in translation and found to localize to starvation stress granules (Zinoviev et al., 2012a; Fritz et al., 2015). A greater association between EIF4E3/EIF4G4 and different RNA helicases may also reflect a greater need for these helicases during translation of mRNAs with longer 5′UTRs and with greater complexity than those found with the mRNAs bound by the EIF4E4/EIF4G3 complex. This is in agreement with the observation that the ribosomal protein mRNAs in *T. brucei*, specifically found with EIF4E4/EIF4G3, have 5′UTRs noticeably shorter than average (Jensen et al., 2014), therefore with presumably less requirement for helicases.

Only recently has a specific mRNA target been identified for one of the trypanosomatid eIF4F-like complexes, the EIF4E6/EIF4G5 subunits. The mRNA encoding the *T. brucei* Variant Surface Antigen (VSG) binds the RNA-binding protein CFP2, which specifically co-precipitates with EIF4E6, EIF4G5 and the translational activators MKT1 and PBP1. CFP2 mediates recruitment of the VSG mRNA by the translation apparatus through its binding to a defined sequence motif within the mRNA’s 3′UTR (Melo do Nascimento et al., 2021). Here, our results define a new set of mRNAs associated with the *T. brucei* EIF4E4/EIF4G3 complex, those encoding ribosomal proteins. This agrees with our recent observations from *Leishmania* which also show the specific co-precipitation of these mRNAs with RBP23 and PABP1, with both proteins also co-precipitating specifically with the EIF4E4/EIF4G3 complex (Assis et al., 2021). The ribosomal protein mRNAs are generally highly expressed, but presumably require tight control of their translation. In mammals they are characterized by the presence of an oligopyrimidine tract at their 5′ ends, with their translation regulated through the binding of the LARP RNA binding protein (Fonseca et al., 2018). In yeast, these mRNAs are also very efficiently translated and are found more closely associated with a closed loop complex formed by eIF4F and PABP, whereas most mRNAs encoding proteins classified in the catalytic activity categories seem to require a more relaxed eIF4F/PABP association for translation efficiency (Costello et al., 2015). In trypanosomatids, the presence of the 5′ SL sequence common to all mRNAs and starting with two purines rules out a mammal-like mode of mRNA recognition for the ribosomal protein mRNAs. These transcripts are markedly absent from cytoplasmic stress granules, which otherwise harbor most other cellular mRNAs (Fritz et al., 2015). Confirmation of their association with the EIF4E4/EIF4G3 complex, first suggested based on data from the characterization of the *T. brucei* PABPs (Zoltner et al., 2018), defines how their translation can be specifically regulated independently from most other mRNAs, as seen in mammals. This regulation may involve phosphorylation of both EIF4E4 and PABP1, a simultaneous event (de Melo Neto et al., 2015, 2018) that is mediated, in part at least, by the cell cycle regulated CRK1 kinase (An et al., 2018), here confirmed to be enriched with both EIF4E4 as well as EIF4G3. Our results then identify a specific translation regulation event which might be linked to the EIF4E4/EIF4G3 complex.

Although EIF4E4 has been shown in *Leishmania* to be essential for survival (de Melo Neto et al., 2015), EIF4E4 knockdown in *T. brucei* procyclic cells did not impact on viability or growth. This may be due to residual EIF4E4 left after the knockdown being enough to support its role in translation, despite not being detectable by western blot, or to EIF4G3 binding to the target mRNAs in the absence of EIF4E4. Another alternative would be for an eIF4E homologue other than EIF4E3, to compensate for the lack of EIF4E4. A possible candidate might be EIF4E1, since RNAi targeting both EIF4E1 and EIF4E4 leads to a drastic reduction in cell growth and translation (Freire et al., 2011). The eIF4Gs were not seen in previous EIF4E1 pull-downs (Falk et al., 2021), but in those experiments the association might have been reduced by detergent. Nevertheless, published evidence so far is more compatible with trypanosome EIF4E1 being a repressor of translation (Erben et al., 2014; Lueong et al., 2016; Terrao et al., 2018; Falk et al., 2021). The question then remains regarding how the *T. brucei* procyclic cells can compensate and survive after such a substantial reduction in EIF4E4 levels, greater than 90% after RNAi (Freire et al., 2011), while being sensitive to very limited changes in the abundance of its EIF4G3 partner (Moura et al., 2015).

The data reported in this manuscript defines, for the first time, specific differences related to the mode of action the two eIF4F-like complexes investigated here, highlighting known and likely binding partners as well as the different mRNA targets. Our data also highlights the need for further investigation into their function, especially when considering the role of the EIF4E3/EIF4G4 subunits, in translation and otherwise, perhaps through the characterization of several of the co-precipitating proteins found here. Knowledge regarding the EIF4E4/EIF4G3 mode of action benefited substantially from the *Leishmania* work focused on the characterization of its RBP23 and PABP1 partners de (de Melo Neto et al., 2018; Assis et al., 2021). The reasons why more proteins are enriched with EIF4E4 than with EIF4E3 will also need to be addressed, although this might reflect a greater abundance for free EIF4E3, leading to reduced ratios for enriched partners. Another major issue that deserves investigation deals with the features in bound mRNAs which define their association with specific complexes; are there specific motifs, or other properties such as length of the UTRs or nucleotide modifications which define the association with the different complexes? Solving these and other questions raised here, for instance regarding the possible involvement of RNA helicases other than EIF4AI with the function of the EIF4E3/EIF4G4 complex, should clarify important features regarding translation initiation in trypanosomatids and how the different eIF4F complexes can help these parasites regulate their response to external stimuli. They might also shed some light on poorly defined mechanistic aspects of translation initiation in eukaryotes in general.

## Data Availability

The datasets presented in this study can be found in online repositories. The names of the repository/repositories and accession number(s) can be found below: https://www.ebi.ac.uk/pride/archive/, PXD033857 https://www.ncbi.nlm.nih.gov/geo/, GSE206064.

## Supplementary material

SUPPLEMENTARY FIGURE S1 Assessment of the immunoprecipitation procedure. Western blot assays confirming the efficiency of the IPs using EIF4E3 and EIF4E4 polyclonal antibodies in *T. brucei* cell lines. Three independent experiments, each with cellular extracts from an independent transgenic clone, were carried out for each immunoprecipitated protein, with corresponding sets of three negative controls performed using only the beads incubated with the same set of extracts.

SUPPLEMENTARY FIGURE S2 Direct comparison of proteins specifically co-precipitated with the two sets of eIF4E and eIF4G homologues from the EIF4E3/EIF4G4 and EIF4E4/EIF4G3 complexes. These results, also detailed in the Supplementary Table S3, highlight the 20 top-most enriched proteins from each of the tagged baits (log2 enrichment values are shown). For clarity the proteins co-precipitated with the two eIF4Es are shown together (top) and separately from those co-precipitated with the two eIF4Gs (bottom), with the proteins found with the EIF4E4/EIF4G3 pair shown inverted in the graphs in comparison with the ones from EIF4E3/EIF4G4. Arrows colored in black generally indicate that the proteins co-precipitating with the corresponding pair are among those shown in Tables 1–4, while the red color indicates those that are also present with the protein pair but with a lower enrichment (enrichment <4 and >2; shown in the Supplementary Table S2).

SUPPLEMENTARY FIGURE S3 Quantification of the aberrant phenotype induced by the EIF4E3 and EIF4E4 RNAi. Results from two sets of experiments are shown, carried out with distinct clones for both EIF4E3 and EIF4E4 RNAi cells.

SUPPLEMENTARY FIGURE S4 mRNA groups associated with EIF4E3 and EIF4E4 from *T. brucei* (SOLID sequencing). mRNA groups associated with EIF4E3 and EIF4E4 from *T. brucei*. Upregulated genes were manually classified and grouped using the gene ontology (GO) terms according to their molecular function. “Upregulated” means at least 2-fold more abundant than in the negative control.
