# Supplementary material for: Distinct mRNA and protein interactomes highlight functional differentiation of major eIF4F-like complexes from Trypanosoma brucei
Source: Front Mol Biosci. 2022 Oct 7;9:971811. doi: 10.3389/fmolb.2022.971811 (PMC9585242; doi:10.3389/fmolb.2022.971811)
Supplement: Supplementary file 3 [file Table1.DOCX]

**Supplementary Table S1**

**Oligonucleotides used for the generation of the transgenic cell lines**

| **Gene** | **Sense** | **Sequence** |
| --- | --- | --- |
| **EIF4E3 Deletion** | ***Foward*** | GTTCTCTAGTTTTCAGGGAGAAGGTAACAGTTGGTAAACCTCCGACATAAGGGAGAGTAGCAAACAGGCAGCGAGGTTAT ATGATTGAACAAGATGGATTG |
|  | ***Reverse*** | CGCCCACCATACATGCACACACACACACACAAAAGAAAAACATGTTCTGTTTACGGTACGTGTACGCAAGCAGCGATTA TCAGAAGAACTCGTCAAGAA |
| **EIF4E4 Deletion** | ***Foward*** | TTACGTAGGAACGAACTAAGCCGTCGTATACTAGTACCGGTGAGGAGGCTGGTAGTGACCTTCATCTGATCGGCAGTGTTG ATGATTGAACAAGATGGATTG |
|  | ***Reverse*** | GCATAAAAACCACTAACCAAAATAAATGAGTGATGGTGTTGGCCGCCTCCTCCACAAGTAACAAAACGCACGCCCTCACGC TCAGAAGAACTCGTCAAGAA |
| **EIF4G3 Deletion** | ***Foward*** | GTGGGGAGGGGAGTACGCCAGAGCGGGAGTGAGGACCATCTCCGATAACGTCGAAGGAAAGGTTGGAGAAGCTGGTGATCA ATGATTGAACAAGATGGATTG |
|  | ***Reverse*** | CACTCTGACGGCGAGACGCCACCAGAGGTGCTGCCAACAACGAACTTCACCCTTCTTACGCTGTTCCCTATCCGCTCACATCAGAAGAACTCGTCAAGAA |
| **EIF4G4 Deletion** | ***Foward*** | CCTCGAGCTGTCCTTCCAGTTGACTTATTTTGATAAGTACATACAAATTTGTATTTATATTACTAGTTTGAGCAGGGGGAA ATGATTGAACAAGATGGATTG |
|  | ***Reverse*** | CAACCTTTCAGCTCTGCAGTGTTCCTGACCAAAGTTTCTCCCTTCACCTACTACCGCTGCCCTCCACAACCTTGCTAGACGTCAGAAGAACTCGTCAAGAAG |
| **EIF4E3 YFP**  **N-terminal** | ***Foward*** | GTTCTCTAGTTTTCAGGGAGAAGGTAACAGTTGGTAAACCTCCGACATAAGGGAGAGTAGCAAACAGGCAGCGAGGTTAT ATGCCTTTGTCTCAAGAAGAA |
|  | ***Reverse*** | CCACCACCGTTTCCACGCCGACCACCGGATCCTGGTGTGCGGTTCCCCTTCGGTACAAATTCCTCCGCTTCTGGATTC ATGGTGGCGGAAGCTTGAGAAC |
| **EIF4E4 YFP**  **N-terminal** | ***Foward*** | TTACGTAGGAACGAACTAAGCCGTCGTATACTAGTACCGGTGAGGAGGCTGGTAGTGACCTTCATCTGATCGGCAGTGTTG ATGCCTTTGTCTCAAGAAGAA |
|  | ***Reverse*** | GGGTGCAGTTACAGCCACCGCCGACGATCTTTTCTGCCATGACGGAGTGTACTCCACCGCATCAGCACGTAAATTTTGC ATGGTGGCGGAAGCTTGAGAAC |
| **EIF4G3 YFP**  **N-terminal** | ***Foward*** | GTGGGGAGGGGAGTACGCCAGAGCGGGAGTGAGGACCATCTCCGATAACGTCGAAGGAAAGGTTGGAGAAGCTGGTGATCA ATGCCTTTGTCTCAAGAAGAA |
|  | ***Reverse*** | CTCAAGTGAAAAGCCGGGGTACGGTGGCTCCGGGTAGAGCGACCGCAGTTCGAGAATTTGGTCGATGGTGTAGACGTGC ATGGTGGCGGAAGCTTGAGAAC |
| **EIF4G4 YFP**  **N-terminal** | ***Foward*** | ACTCATAGGGATAAGAAGGGAGGAAGCAACAGAGCAAGGTGATAGGATTTTTTTTTTTGAAACGGGGAGACGTGTGTAATAATGCCTTTGTCTCAAGAAGAA |
|  | ***Reverse*** | AAGATCCGACACAGACATTAGCCGCGACCCGCCAGCATATCTTGGATCGTTGCTGGTGACACCACGAGGTTTGAACAGC ATGGTGGCGGAAGCTTGAGAAC |
