# Supplementary figures and images for: Distinct mRNA and protein interactomes highlight functional differentiation of major eIF4F-like complexes from Trypanosoma brucei
Source: Front Mol Biosci. 2022 Oct 7;9:971811. doi: 10.3389/fmolb.2022.971811 (PMC9585242; doi:10.3389/fmolb.2022.971811)

## Bezerra et al., Suppl. Figure 4

### Bound mRNAs

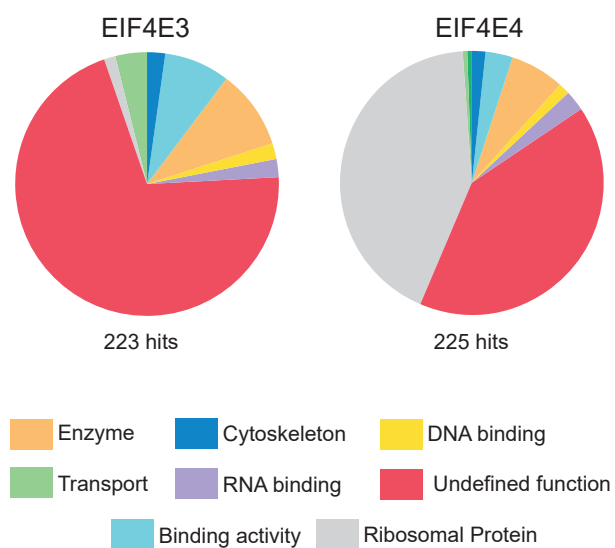

Supplement: Supplementary file 4 [file Image4.PDF]

Bezerra et al., Suppl. Figure 2

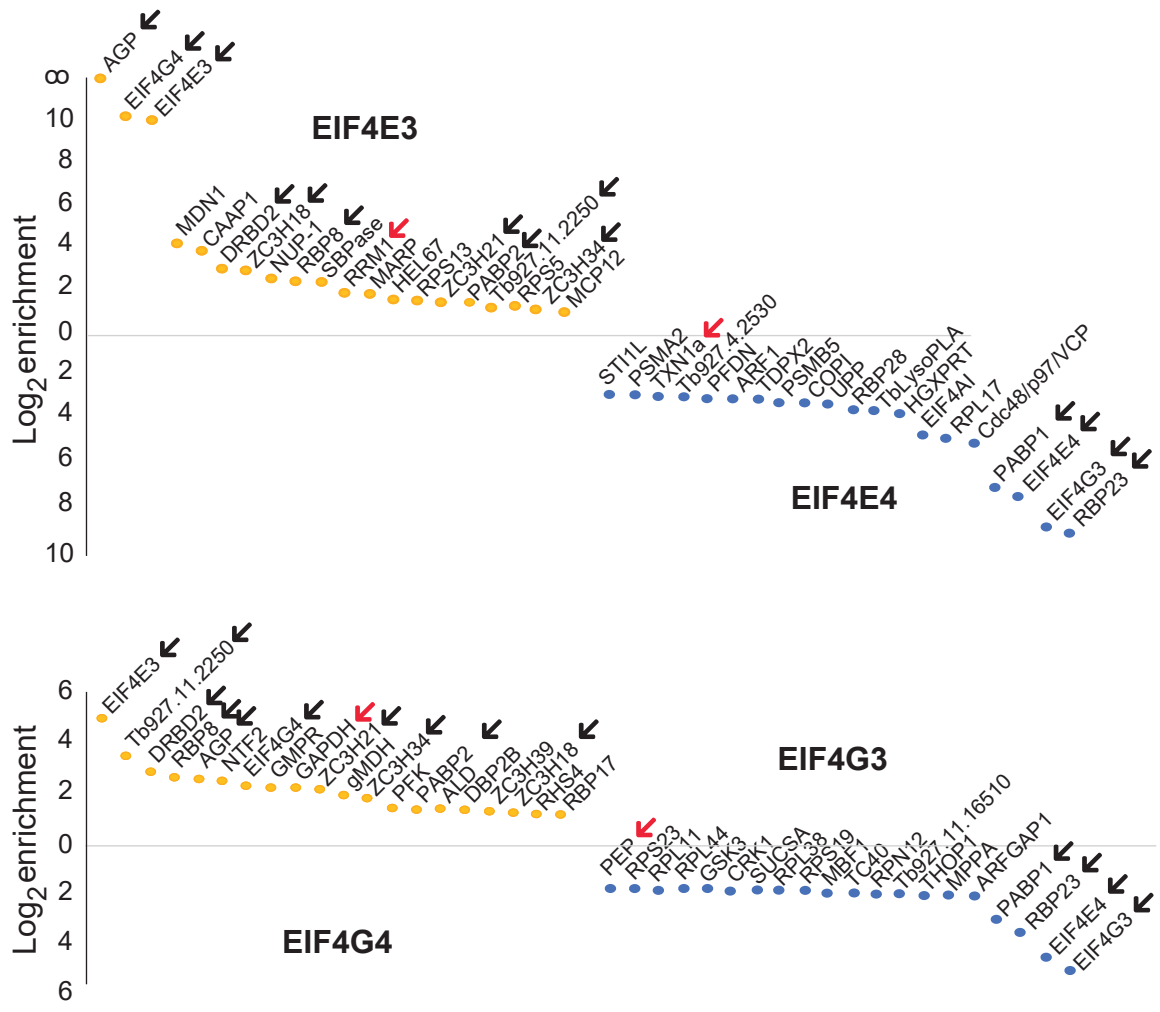

Supplement: Supplementary file 5 [file Image2.PDF]

Bezerra et al., Suppl. Figure 1

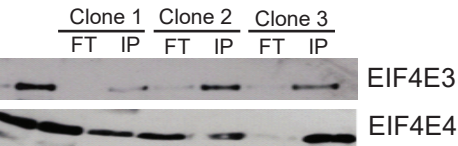

Supplement: Supplementary file 12 [file Image1.PDF]
