# Supplementary material for: Distinct mRNA and protein interactomes highlight functional differentiation of major eIF4F-like complexes from Trypanosoma brucei
Source: Front Mol Biosci. 2022 Oct 7;9:971811. doi: 10.3389/fmolb.2022.971811 (PMC9585242; doi:10.3389/fmolb.2022.971811)
Supplement: Supplementary file 7 [file Image3.PDF]

# Bezerra et al., Suppl. Figure 3

| Days after RNAi induction | EIF4E3 Clone 1 | | EIF4E3 Clone 2 | | EIF4G4 Clone 1 | | EIF4G4 Clone 2 | |
|---|---|---|---|---|---|---|---|---|
| | Normal Phenotype (%) | Aberrant Phenotype (%) | Normal Phenotype (%) | Aberrant Phenotype (%) | Normal Phenotype (%) | Aberrant Phenotype (%) | Normal Phenotype (%) | Aberrant Phenotype (%) |
| 1 | 99 | 1 | 97 | 3 | 92 | 8 | 89 | 11 |
| 2 | 91 | 9 | 95 | 5 | 51 | 49 | 58 | 42 |
| 3 | 73 | 27 | 67 | 23 | 36 | 64 | 61 | 39 |
| 4 | 61 | 39 | 67 | 33 | 42 | 58 | 59 | 41 |
| 5 | 70 | 30 | 67 | 33 | 33 | 67 | 35 | 65 |
